# LRG1 inhibition promotes acute pancreatitis recovery by inducing cholecystokinin Type 1 receptor expression via Akt

**DOI:** 10.7150/thno.110116

**Published:** 2025-03-18

**Authors:** Seok Ting Lim, Xinmei Zhao, Shuqing Liu, Wenjuan Zhang, Yuanyang Tan, Nidula Mullappilly, Sandip M Swain, Mei Ling Leong, Ravisankar Rajarethinam, Kah Fei Wan, Christiane Ruedl, Rodger A. Liddle, Liang Li, Xiaomeng Wang

**Affiliations:** 1 Centre for Vision Research, Duke-NUS Medical School, Singapore.; 2 Singapore Eye Research Institute, Singapore.; 3 Guangdong Provincial Key Laboratory of Gastroenterology, Department of Gastroenterology, Nanfang Hospital, Southern Medical University, No. 1838 North Guangzhou Avenue, Guangzhou, China.; 4 School of Medical Technology, Beijing Institute of Technology, 100081, Beijing, China.; 5 Department of Medicine, Duke University, Durham, NC, 27710, U.S.A.; 6 School of Biological Sciences, Nanyang Technological University, Singapore.; 7 Advanced Molecular Pathology Laboratory (AMPL), Institute of Molecular and Cell Biology, A*STAR, Singapore.; 8 Antibody Technology Division, Experimental Drug Development Centre, A*STAR, Singapore.; 9 Department of Pharmacology, Joint Laboratory of Guangdong-Hong Kong Universities for Vascular Homeostasis and Diseases, School of Medicine, Southern University of Science and Technology.; 10 Institute of Molecular and Cell Biology, A*STAR, Singapore.

**Keywords:** Acute pancreatitis, Leucine-rich alpha-2 glycoprotein 1, Cholecystokinin Type 1 receptor, Transforming-growth factor-beta, Pancreatic regeneration

## Abstract

**Rationale:** Acute pancreatitis (AP) is a common gastrointestinal disease affecting nearly 3 million people annually worldwide. Although AP is typically self-limiting, up to 20% of patients may develop life-threatening complications. Individuals who suffer from AP also have an increased likelihood of developing other exocrine and endocrine pancreatic disorders. However, to date, there are no specific, targeted treatment modalities that can effectively improve the clinical outcomes of AP.

Leucine-rich alpha-2 glycoprotein 1 (LRG1) is a multifunctional protein with established roles in inflammation and cell mitosis. This study aims to investigate the functional role of LRG1 in AP progression and develop LRG1-targeted AP therapeutics.

**Methods:** Levels of circulating and tissue LRG1 were determined in human patient samples and mouse models of caerulein-induced AP and pancreatic duct ligation-induced AP. Histopathological grading, amylase assay, real-time polymerase chain reaction analysis and Western blotting were used to evaluate the extent of pancreatic damage and recovery following caerulein-induced AP in both wild-type and *Lrg1^-/-^* mice. Primary acinar cells were also isolated from mice for in-vitro mechanistic studies. LRG1 neutralizing antibody was administered post-AP induction to evaluate its therapeutic potential in improving AP outcomes.

**Results:** LRG1 is markedly increased in serum and acinar cells of AP patients and C57BL/6 mice subjected to caerulein-induced AP or pancreatic duct ligation-induced AP. Despite demonstrating no obvious pancreatic dysfunction, *Lrg1^-/-^* mice exhibited more severe pancreatic damage and inflammation during the early stages of caerulein-induced AP. However, the resolution of AP was accelerated in the absence of Lrg1, which is at least partially due to LRG1's role in regulating the expression of trophic cholecystokinin (CCK) Type 1 receptor (CCK1R) via the TGFβ/ALK5/AKT pathway in acinar cells. Importantly, the administration of an LRG1-blocking antibody promoted AP recovery, evidenced by reduced overall inflammation and increased acinar cell proliferation.

**Conclusions:** Our data provide compelling evidence for targeting LRG1 as a potential innovative therapy for promoting AP recovery.

## Introduction

Acute pancreatitis (AP) affects 30-40 individuals per 100,000 population worldwide annually and is a common cause of hospitalization for gastrointestinal-related diseases 1. Although the majority of AP patients experience mild symptoms that resolve with simple supportive measures, 20% may develop life-threatening systemic complications such as sepsis, systemic inflammatory response syndrome (SIRS), multiorgan dysfunction syndrome (MODS) 2, with mortality ranging from 8%-39% 3. Importantly, AP survivors often face an increased risk of developing recurrent and chronic adverse events, including new-onset diabetes, exocrine pancreatic insufficiency, chronic pancreatitis, and pancreatic cancer 4. Conventional interventions are primarily supportive and nonspecific, focusing on alleviating symptoms 5 rather than preventing the progression or decreasing the severity of AP. To date, there are no specific, targeted treatment modalities that effectively improve the clinical outcomes of AP.

Irrespective of the etiology, AP is characterized by necro-inflammatory changes predominantly affecting the digestive enzyme-producing acinar cells 6. The initial pancreatic damage leads to increased production of inflammatory cytokines and chemokines, such as tumour necrosis factor-alpha (TNF-α) and interleukin (IL) 1β, 6, and 18, which enhance the recruitment of leukocytes to the injury site 7, further amplifying the initial pro-inflammatory response. Although infiltrated leukocytes are critical for clearing injured acinar cells, persistent inflammation can extend to other organs, causing SIRS and MODS 8. Several anti-inflammatory cytokines and specific cytokine inhibitors have been tested in preclinical models and clinical trials, demonstrating efficacy in suppressing systemic inflammatory reactions in AP 9, 10. However, compensatory anti-inflammatory response syndrome (CARS) is concomitantly associated with systemic inflammation in AP and is a prerequisite for wound healing 11, 12. As AP patients are typically hospitalized at a late stage of disease development, CARS may have developed 13, 14, making immune modulation therapy potentially risky by rendering the host susceptible to secondary infections and worsening the prognosis of AP.

Animal studies have shown that the pancreas retains a surprising capacity for regeneration following AP 15, 16. Therefore, strategies to enhance acinar cell regeneration may be best suited for clinical intervention to limit the progression of AP and improve disease outcomes. Leucine-rich alpha 2-glycoprotein 1 (LRG1) is an acute-phase protein that rapidly increases after microbial or other inflammatory stimuli 17. Intriguingly, LRG1 shares sequence similarity with the apoptotic peptidase activating factor, Apaf-1 and competes with Apaf-1 for binding to cytochrome C (Cyt C) 18. In doing so, LRG1 serves as an effective trap to sequester Cyt C released from apoptotic cells and, therefore, protects cells against its toxic effects, eventually, favouring their survival. In addition to protecting cells through clearing pro-apoptotic factors like Cyt C, LRG1 is mitogenic by regulating multiple signalling pathways 19-22. An anti-proliferative role of LRG1 has also been reported in osteoporosis 23 and keratinocytes 24. To date, the role of LRG1 in pancreatic cells and AP remains to be elucidated.

In the current study, we found that LRG1 was expressed at high levels in the serum of newly admitted AP patients. Consistent with this finding, LRG1 was markedly induced in the circulation and pancreatic tissue of C57BL/6 mice subjected to caerulein-induced or pressure-induced AP. Although multiple cell types contribute to elevated LRG1 levels in AP, non-myeloid cell-derived LRG1 plays a fundamental role in AP pathogenesis. Interestingly, both local and systemic LRG1 levels positively correlated with IL-6 in AP. We further showed that LRG1 served as a direct target gene of the IL-6/STAT3 pathway and LRG1 regulated the expression of the trophic gene CCK1R by regulating the non-canonical TGFβ/Akt signalling in acinar cells, which not only explains the more severe pancreatic injury following caerulein-induced AP but also the accelerated recovery of AP in *Lrg1^-/-^* mice. Finally, our work demonstrated increased CCK1R expression, acinar cell proliferation, and AP recovery in C57BL/6 mice subjected to treatment with LRG1 blocking antibody. Together, our study demonstrated, a critical role of LRG1 in AP recovery, which could underpin the development of more effective treatments for AP, including the prevention of severe and chronic pancreatitis.

## Results

### LRG1 is not required for normal pancreatic structure and function

**Figure 1:**
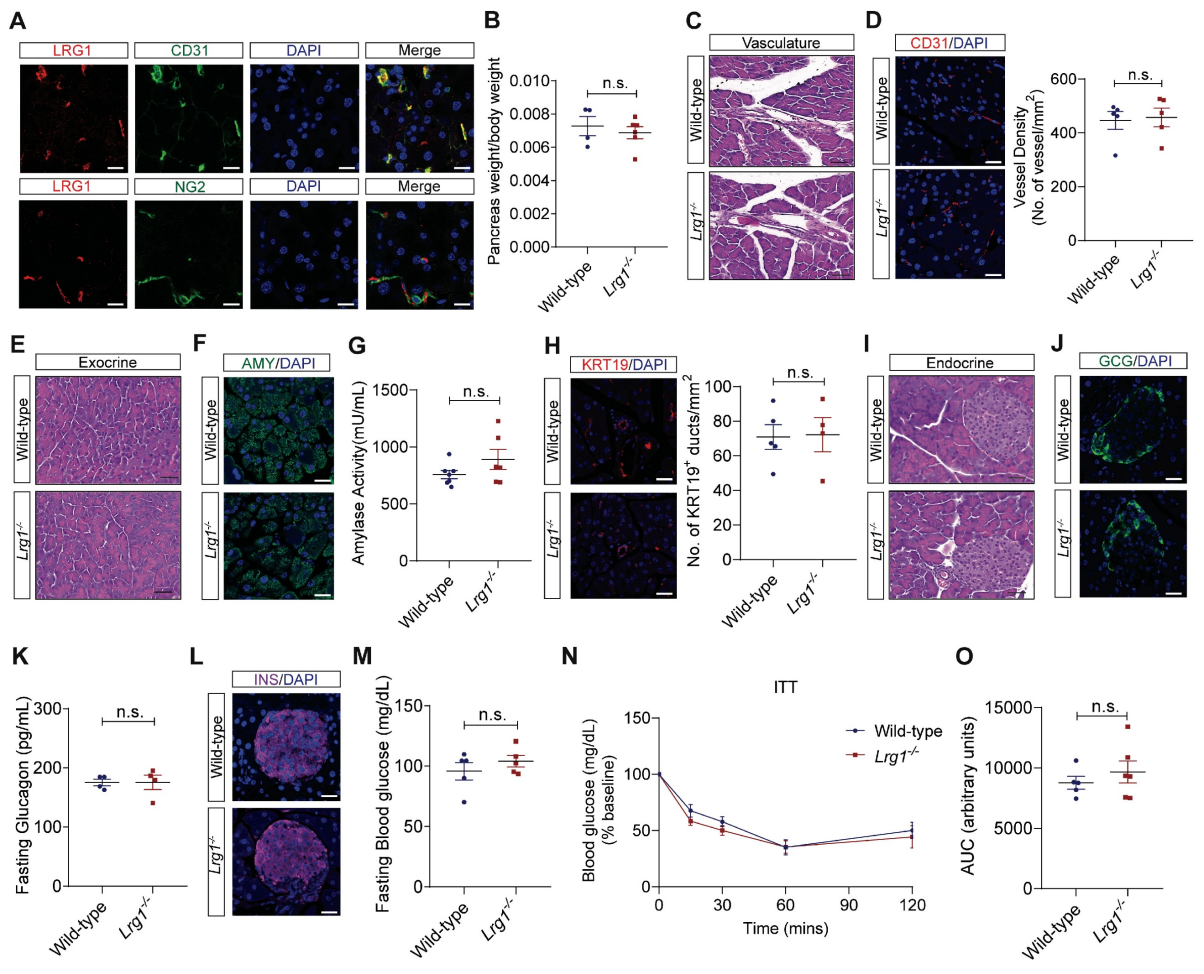
** Loss of LRG1 does not affect the structure and function of the normal mouse pancreas. (A)** Immunofluorescence staining of LRG1 (red), CD31 or NG2 (green), and DAPI (blue) in the normal mouse pancreas. **(B)** Pancreas weight to body weight ratio of wild-type and *Lrg1^-/-^* mice. **(C)** H&E staining showing vasculature of wild-type and *Lrg1^-/-^* mouse pancreas. **(D)** Immunofluorescence staining of CD31 (red) and DAPI (blue) (left) and quantification of vessel density (right) in wild-type and *Lrg1^-/-^* mouse pancreas. **(E)** H&E staining showing exocrine component of wild-type and *Lrg1^-/-^* mouse pancreas. **(F)** Immunofluorescence staining of amylase (AMY, green) and DAPI (blue) in wild-type and *Lrg1^-/-^* pancreas. **(G)** Serum amylase activity in wild-type and *Lrg1^-/-^* mice. **(H)** Immunofluorescence staining of cytokeratin 19, KRT19 (red), and DAPI (blue) (left) and quantification of ductal density (right) in wild-type and *Lrg1^-/-^* pancreas. **(I)** H&E staining showing endocrine component of wild-type and *Lrg1^-/-^* mouse pancreas. **(J)** Immunofluorescence staining of glucagon (GCG, green) and DAPI (blue) in wild-type and *Lrg1^-/-^* pancreas. **(K)** Fasting glucagon levels in wild-type and *Lrg1^-/-^* mice. **(L)** Immunofluorescence staining of insulin (INS, magenta) and DAPI (blue) in wild-type and *Lrg1^-/-^* pancreas. **(M)** Fasting blood glucose levels in wild-type and *Lrg1^-/-^* mice following the intraperitoneal delivery of insulin. Intraperitoneal insulin tolerance (ITT) is indicated as **(N)** the percentage of basal glucose and **(O)** the area under the curve (AUC). All images are representative, scale bar: 50μm for H&E images and 20μm for immunofluorescence images. Data are presented as mean ± s.e.m. Significance was determined by unpaired, two-tailed Student's t-test of n ≥ 4 mice; n.s.: not significant.

Previous studies have shown that LRG1 is expressed in the vasculature of various organs in both mice and humans 20, 25. Although the pancreas is a highly vascularized organ 26, the expression pattern of LRG1 and its function in normal pancreas remain to be elucidated. Immunofluorescence staining revealed that Lrg1 was highly enriched in CD31^+^ pancreatic endothelial cells but not in NG2^+^ perivascular cells in normal mouse pancreas (**Figure 1A**). Despite its expression in normal pancreatic vasculature, *Lrg1* deletion did nowt affect the weight (**Figure 1B**), vessel morphology (**Figure 1C**), and vessel density (**Figure 1D**) of the pancreas in adult mice. Consistent with these observations, the expression levels of vascular endothelial growth factor A (*Vegfa*) and its receptor *Vegfr2* were comparable in the pancreas of *Lrg1^-/-^* and wild-type mice (**Supplementary Figure S1A and B**). The pancreas comprises two morphologically distinct tissues: the exocrine pancreas, dominated by highly specialized zymogen/enzyme secreting acinar cells and the ductal network, and the endocrine pancreas, organized into islets of Langerhans responsible for secreting hormones such as glucagon and insulin 26. Hematoxylin and eosin (H&E) staining of the exocrine compartment (**Figure 1E**) and immunofluorescence staining for amylase demonstrated no obvious change in the density and organization of acinar cells in the *Lrg1^-/-^* pancreas (**Figure 1F**). Supporting this observation, the mRNA levels of pancreatic alpha-amylase (*Amy2*) in *Lrg1^-/-^* mice were comparable to those in wild-type controls (**Supplementary Figure S1C**). Blood amylase levels, which reflect the integrity and function of the exocrine pancreas 27, were also unaffected by *Lrg1* ablation (**Figure 1G**). To further understand Lrg1's role in the exocrine pancreas, we quantified the number of cytokeratin 19 (KRT19)^+^ ductal cells 28 (**Figure 1H**) and mRNA levels of *Krt19* (**Supplementary Figure S1D**) finding no significant differences in the absence of *Lrg1*. The impact of *Lrg1* deletion on the structure and function of the endocrine pancreas was also evaluated. No morphological difference was observed in the pancreatic islets as demonstrated by H&E staining (**Figure 1I**). Immunofluorescence staining showed that glucagon-producing α cells were distributed on the rim of islets in both wild-type and *Lrg1^-/-^* mice (**Figure 1J**). Consistently, pancreatic glucagon (*Gcg*) mRNA levels (**Supplementary Figure S1E**) and fasting glucagon levels (**Figure 1K**) were not affected by *Lrg1* deletion. Similarly, the distribution of insulin-producing β cells (**Figure 1L**), pancreatic insulin (*Ins*) gene expression levels (**Supplementary Figure S1F**), and fasting blood glucose levels (**Figure 1M**) were comparable in *Lrg1^-/-^* and control mice. To investigate insulin sensitivity, *Lrg1^-/-^* and control mice were subjected to intraperitoneal injection of insulin*.* As expected, blood glucose levels were markedly reduced following insulin administration (**Figure 1N**) in both *Lrg1^-/-^* and control mice. The area under the curve (AUC) analysis indicated that *Lrg1* deletion did not affect insulin action (**Figure 1O**). Additionally, mRNA analysis also revealed no differences in gene expression levels of inflammatory cytokines, *Tnfa* (**Supplementary Figure S1G**), *Il6* (**Supplementary Figure S1H**), *Cxcl1* (**Supplementary Figure S1I**) and *Il1β* (**Supplementary Figure S1J**) in both wild-type and *Lrg1^-/-^* pancreas. Together, our data show that *Lrg1* deletion had no impact on the structure and function of both the exocrine and endocrine pancreas under physiological conditions.

### LRG1 is strongly induced in AP

**Figure 2:**
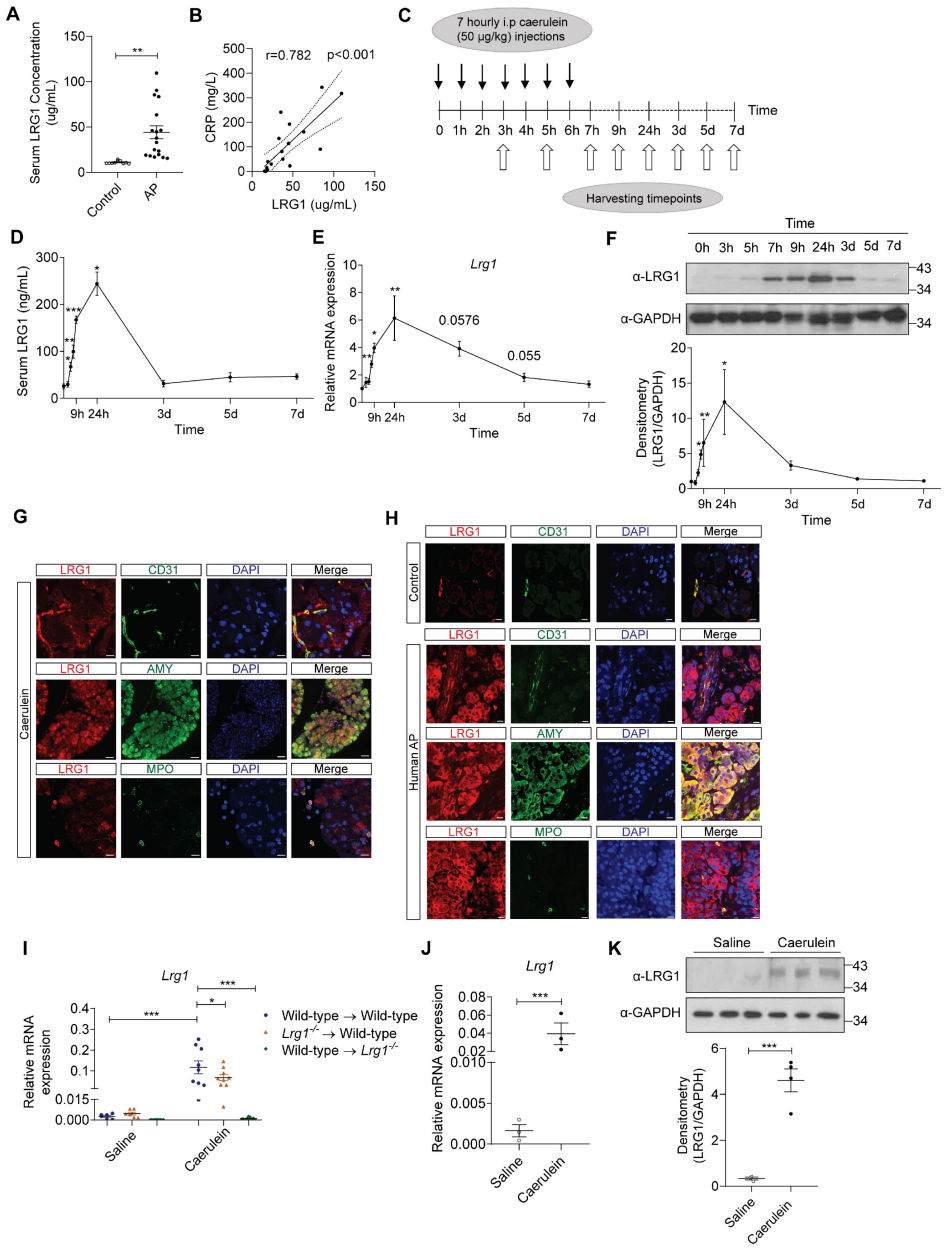
** LRG1 is highly induced in humans and mice during AP. (A)** ELISA analysis of LRG1 levels in the serum of healthy controls (n = 10) and AP patients (n = 18). **(B)** Correlation analysis with regression line (95% confidence intervals) of serum LRG1 and CRP in AP patients.** (C)** Schematic diagram of caerulein-induced AP in mice. **(D)** ELISA analysis of serum LRG1 levels in mice subjected to caerulein-induced AP. **(E)** qRT-PCR analysis of pancreatic *Lrg1* levels at various time points during AP progression. **(F)** Western blot (top) and densitometry analysis (bottom) of pancreatic LRG1 levels at various time points during AP progression. **(G)** Immunofluorescent staining of LRG1 (red), CD31 or AMY or MPO (green), and DAPI (blue) in mouse pancreas 24 hours following AP. Scale bar (CD31 and MPO): 10μm, Scale bar (AMY): 40μm. **(H)** Immunofluorescent staining of LRG1 (red), CD31 or AMY or MPO (green), and DAPI (blue) in control or human AP pancreas. Scale bar: 10μm. **(I)** qRT-PCR analysis of *Lrg1* in the pancreas of saline or caerulein-treated wild-type recipient mice transplanted with wild-type or *Lrg1^-/-^* BMCs and *Lrg1^-/-^* recipient mice transplanted with wild-type BMCs 24 hours post AP induction. **(J)** qRT-PCR or **(K)** Western blot (top) and densitometry analysis (bottom) of Lrg1 in isolated acinar cells 24 hours following the first caerulein injection. All images are representative. Data are presented as mean ± s.e.m. Significance was determined by one-way ANOVA followed by Holm-Sidak's multiple comparison test or unpaired, two-tailed Student's t-test of n ≥ 3 mice or independent experiments unless stated otherwise; *: p < 0.05, **: p < 0.01, ***: p < 0.001.

Increased LRG1 expression was previously reported in patients with infectious and autoimmune disorders 29-31. However, the association between LRG1 and AP has not been reported. We found that serum LRG1 levels were significantly elevated in individuals with AP compared to healthy controls (**Figure 2A**) and were closely associated with C-reactive protein (CRP) (**Figure 2B**), neutrophil levels (**Supplementary Figure S2A**) and serum lipase (**Supplementary Figure S2B**), important biomarkers for predicting AP severity, SIRS and MODS 32, 33. As the events of AP progression are inaccessible for direct observation in humans, animal models are commonly used to study disease pathophysiology and evaluate novel therapeutics. Caerulein, a decapeptide structurally related to the C-terminus of cholecystokinin (CCK), regulates the synthesis and secretion of digestive enzymes, including chymotrypsinogen and amylase, in pancreatic acinar cells 34. C57BL/6 mice subjected to repetitive injections of caerulein (**Figure 2C**) develop key pathologic events of AP observed in humans 35, such as acinar cell apoptosis and pancreatic edema (**Supplementary Figure S2C**), hyperamylasemia (**Supplementary Figure S2D**), and infiltration of inflammatory cells in the injured pancreas (**Supplementary Figure S2E**). Consequently, *Amy2* mRNA levels were significantly reduced in C57BL/6 mice following the induction of AP (**Supplementary Figure S2F**), an indication of pancreatic acinar cell damage. On the other hand, the endocrine pancreas remained unaffected by caerulein treatment, as evidenced by the preserved organization of insulin- and glucagon-producing cells compared to controls (**Supplementary Figure S2G**). Next, we assessed serum (**Figure 2D**) and pancreatic (**Figures 2E and 2F**) LRG1 levels at various stages of the AP progression in caerulein-treated C57BL/6 mice. Our findings revealed that both circulating and local LRG1 levels peaked 24 hours following the initial caerulein injection, aligning with the stage of the maximum pancreatic injury 36. LRG1 levels declined gradually thereafter, returning to baseline by day 3 in the circulation and by day 5 in the pancreas. Besides the vasculature, LRG1 was induced in amylase^+^ acinar cells and myeloperoxidase (MPO)^+^ myeloid cells in the pancreas of mice 24 hours after the initial caerulein treatment, as demonstrated by immunofluorescence (**Figure 2G**) and immunohistochemistry staining (**Supplementary Figure S2H**). These observations were corroborated in a mouse model of pancreatic duct ligation (PDL)-induced AP, which mimics pressure-induced gallstone pancreatitis in humans. Substantial acinar cell death and inflammatory cell infiltration, hallmarks of AP, were evident 24 hours after PDL (**Supplementary Figure S3A**), which was accompanied by concurrent increases in serum (**Supplementary Figure S3B**) and pancreatic LRG1 mRNA (**Supplementary Figure S3C**) and protein (**Supplementary Figure S3D**) levels. Furthermore, in addition to its expression in CD31^+^ endothelial cells, LRG1 was markedly upregulated in amylase^+^ acinar cells and MPO^+^ myeloid cells following PDL-induced AP (**Supplementary Figure S3E**), as well as in AP patients compared to controls (**Figure 2H**). To identify key LRG1-producing cells (haematopoietic versus non-haematopoietic) in the pancreas of AP mice, we performed allogeneic bone marrow transplantation (BMT) in lethally irradiated wild-type and *Lrg1^-/-^* mice (**Figure 2I**). As anticipated, *Lrg1* mRNA levels were significantly elevated in the pancreas of wild-type mice transplanted with wild-type bone marrow cells (BMCs) 24 hours after the first caerulein treatment. Unexpectedly, caerulein treatment also significantly increased pancreatic Lrg1 levels in wild-type mice transplanted with *Lrg1^-/-^* BMCs, though to a lesser extent. Conversely, caerulein did not induce pancreatic Lrg1 expression in *Lrg1^-/-^* recipient mice transplanted with wild-type BMCs. Consistent with these observations, *Lrg1* mRNA (**Figure 2J**) and protein (**Figure 2K**) levels were significantly higher in primary acinar cells isolated from wild-type mice 24 hours following the first caerulein injection, compared to cells isolated from saline-injected controls. These data suggest that the non-haematopoietic cell fraction, likely the pancreatic acinar cells, serves as a major source of LRG1 following caerulein-induced AP.

### IL-6 induces the expression of *Lrg1* in pancreatic acinar cells through the activation of the STAT3 signalling pathway

**Figure 3:**
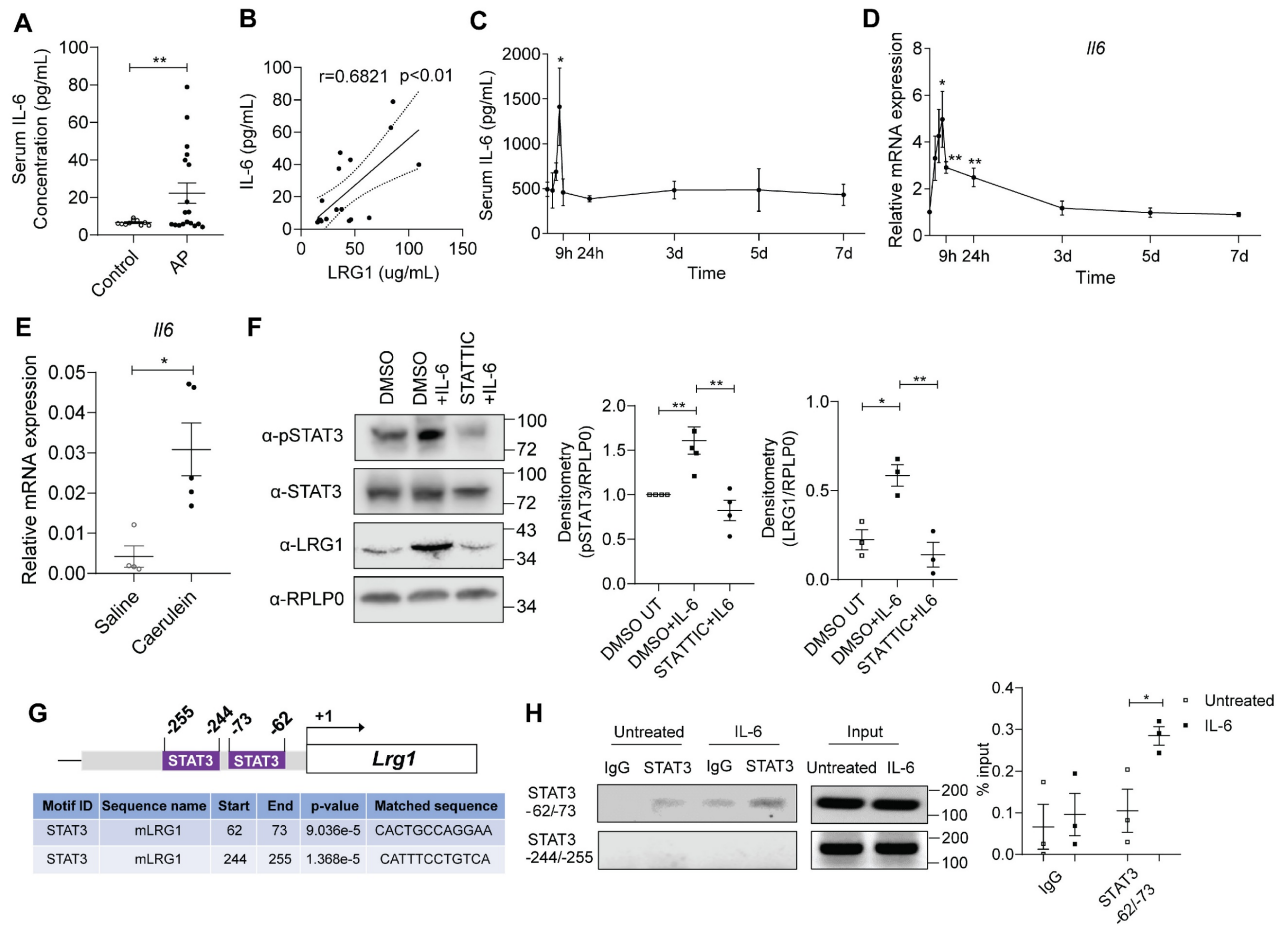
** LRG1 is regulated by IL-6 in pancreatic acinar cells during AP. (A)** ELISA analysis of IL-6 levels in the serum of healthy controls (n = 10) and AP patients (n = 18). **(B)** Correlation analysis with regression line (95% confidence intervals) of serum LRG1 and IL-6 in AP patients.** (C)** ELISA analysis of serum IL-6 levels in mice subjected to caerulein-induced AP. **(D)** qRT-PCR analysis of *Il6* in mouse pancreas at various time points during AP progression. **(E)** qRT-PCR analysis of *Il6* in isolated acinar cells 24 hours following the first caerulein injection. **(F)** Western blot (left) and densitometry analysis (right) of phosphorylated and total STAT3, and LRG1 in wild-type acinar cells subjected to DMSO or STATTIC treatment with or without addition of IL-6. **(G)** Schematic indicating organization of the mouse LRG1 promoter containing two putative STAT3 transcription factor binding sites. **(H)** DNA agarose gel (left) and quantitative analysis (right) of chromatin immunoprecipitation assay for STAT3 and LRG1 promoter association in the presence or absence of IL-6 in primary acinar cells. All images are representative. Data are presented as mean ± s.e.m. Significance was determined by one-way ANOVA followed by Holm-Sidak's multiple comparison test or unpaired, two-tailed Student's t-test of n ≥ 3 mice or independent experiments unless stated otherwise; *: p < 0.05, **: p < 0.01.

IL-6 is an inflammatory cytokine that is closely associated with AP 37, 38. IL-6 levels are elevated in the serum of AP patients (**Figure 3A**) and significantly correlated to serum LRG1 levels (**Figure 3B**). Similarly, serum (**Figure 3C**) and pancreas (**Figure 3D**) IL-6 levels were induced in caerulein-treated C57BL/6 mice, mirroring that of LRG1. Similar to *Lrg1*, *Il-6* was also markedly induced in primary acinar cells isolated from caerulein-treated mice as compared to that in vehicle-treated controls (**Figure 3E**). IL-6 was previously reported to regulate LRG1 expression in non-acinar cells *via* canonical JAK/STAT3 signalling pathway 17, 39. Here we showed that IL-6 promotes STAT3 phosphorylation and *Lrg1* expression in acinar cells, whereas the STAT3-specific inhibitor Stattic significantly attenuated this effect (**Figure 3F**).

*In silico* analysis of the mouse *LRG1* promoter using the HOmo sapiens COmprehensive MOdel COllection (HOCOMOCO) database 40 revealed the presence of two putative STAT3 binding sites, located at positions -62 to -73 and -244 to -255, respectively (**Figure 3G**). Chromatin Immunoprecipitation (ChIP) assay confirmed that IL-6 promotes the binding of STAT3 to the site at -62 to -73, but not to the site at -244 to -255 on the LRG1 promoter (**Figure 3H**). These finding indicate that LRG1 is a direct target gene of IL-6 in pancreatic acinar cells, regulated through the STAT3 signalling pathway.

### Loss of *Lrg1* exacerbates caerulein-induced AP

**Figure 4:**
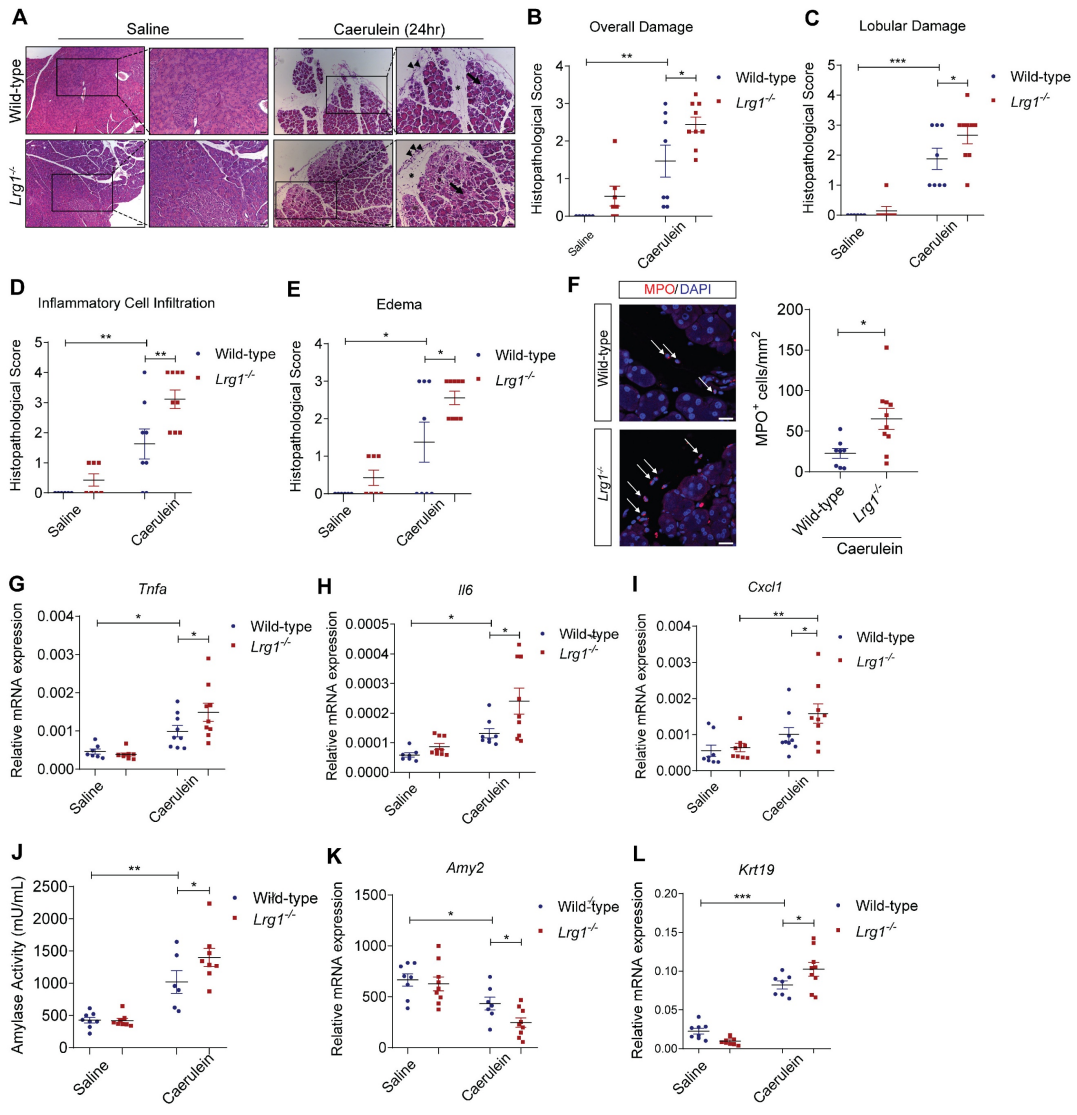
** LRG1 deficiency exacerbates caerulein-induced AP. (A)** H&E images demonstrating the overall pancreatic damage in saline or caerulein-treated wild-type and *Lrg1^-/-^* mice (Inter- and intralobular damage (asterisk), infiltrated inflammatory cells (arrowhead), and edema (arrow)). Scale bar: 50μm, scale bar for boxed regions: 25μm. Histopathological grading of **(B)** overall pancreatic damage **(C)** lobular damage, **(D)** inflammatory cell infiltration, and **(E)** edema in H&E-stained wild-type and *Lrg1^-/-^* pancreatic tissues. **(F)** Immunofluorescent staining against MPO (red) and DAPI (blue) (left) and quantification (right) of MPO^+^ inflammatory cells (arrow) in wild-type and *Lrg1^-/-^* pancreas. Scale bar: 20μm. qRT-PCR analysis of the mRNA levels of inflammatory cytokines, **(G)** *Tnfa*, **(H)** *Il6,* and **(I)** *Cxcl1* in wild-type and *Lrg1*-deficient pancreas. **(J)** Analysis of serum amylase activity in wild-type and *Lrg1^-/-^* mice. qRT-PCR analysis of mRNA levels of pancreatic **(K)** *Amy2* and **(L)** *Krt19*. All analyses were performed 24 hours after the induction of AP. Images are representative. Data are presented as the mean ± s.e.m. Significance was determined by one-way ANOVA followed by Holm-Sidak's multiple comparison test or unpaired, two-tailed Student's t-test of n ≥ 6 mice; *: p < 0.05, **: p < 0.01, ***: p < 0.001.

Having established the positive association between LRG1 and AP, as well as the transcriptional control of LRG1 by IL-6 in pancreatic acinar cells, we next investigated the functional role of *Lrg1* in AP development and progression *in vivo*. Hematoxylin and eosin (H&E) staining (**Figure 4A**), along with histopathological scoring (**Figure 4B**) by a certified pathologist showed significantly worse overall pancreatic injury in *Lrg1^-/-^* mice compared to wild-type controls 24 hours post-AP induction. Specifically, *Lrg1* deletion caused marked disruption of lobular integrity as highlighted by increased inter- and intralobular spaces (**Figure 4C**), heightened infiltration of inflammatory cells (**Figure 4D**), and worse interstitial edema (**Figure 4E**). Immunofluorescence staining further confirmed these observations by showing a substantial increase in myeloperoxidase (MPO)^+^ myeloid cell infiltration (**Figure 4F**) and elevated levels of inflammatory cytokines and chemokines, including *Tnfa* (**Figure 4G**), *Il6* (**Figure 4H**), *Cxcl1* (**Figure 4I**), *Il1β* (**Supplementary Figure S4A**), and *Ccl2* (**Supplementary Figure S4B**), in the pancreas of *Lrg1^-/-^* mice following the induction of AP as compared to wild-type counterparts. In addition, serum amylase activity, a marker of AP severity, was notably higher (**Figure 4J**), while the expression of the pancreatic acinar cell marker *Amy2* (**Figure 4K**) was significantly downregulated, and the ductal marker *Krt19* was induced (**Figure 4L**) in the absence of *Lrg1*. Consistently, key signalling pathways involved in AP pathogenesis, including protein kinase C (PKC), Janus kinase/signal transducers and activators of transcription (JAK/STAT), and c-Jun N-terminal Kinase (JNK) signalling pathways 41, were also affected. Our studies revealed elevated levels of phosphorylated PKCδ (pPKCδ) and PKCε (pPKCε), STAT3 (pSTAT3), and JNK (pJNK) in the pancreas of *Lrg1^-/-^* mice (**Supplementary Figure S4C**). Collectively, these results highlight the critical role of LRG1 in modulating AP pathophysiology.

### Myeloid cell-derived LRG1 is dispensable for AP pathogenesis

**Figure 5:**
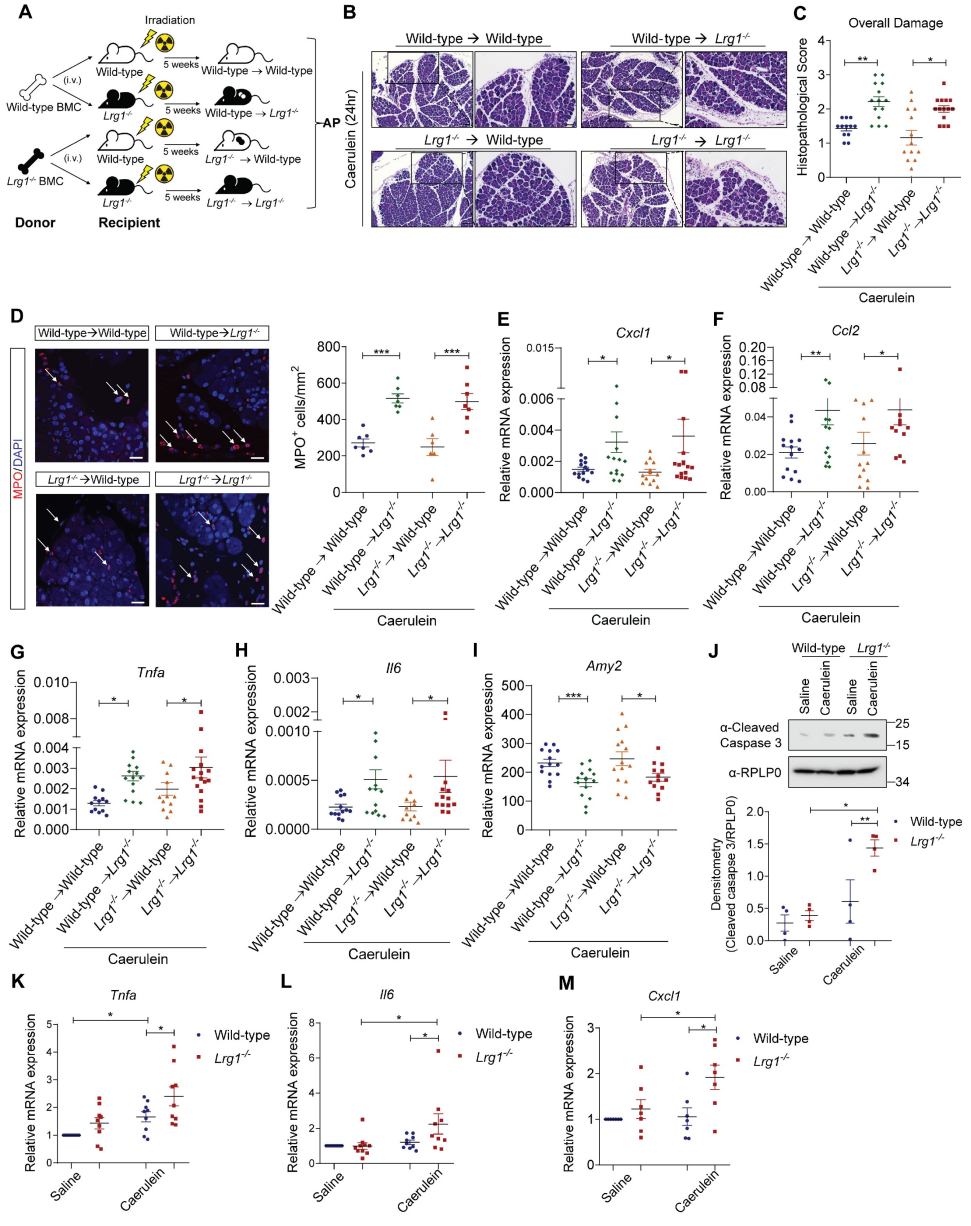
** Non-myeloid cell-derived LRG1 protects against AP-induced damage. (A)** Schematic diagram of bone marrow transplantation with wild-type mice reconstituted with wild-type BMCs (Wild-type → Wild-type), *Lrg1^-/-^* mice reconstituted with wild-type BMCs (Wild-type → *Lrg1^-/-^*), wild-type mice reconstituted with *Lrg1^-/-^* BMCs (*Lrg1^-/-^* → Wild-type) and *Lrg1^-/-^* mice reconstituted with *Lrg1^-/-^* BMCs (*Lrg1^-/-^* →* Lrg1^-/-^*). **(B)** H&E staining and **(C)** histopathological scoring of overall pancreatic damage. Scale bar: 100μm, scale bar of boxed region: 50μm. **(D)** Immunofluorescent staining against MPO (red) and DAPI (blue) (left) and quantification (right) of infiltrated MPO^+^ inflammatory cells (arrow) in the pancreas. Scale bar: 25μm. qRT-PCR analysis of mRNA levels of **(E)** *Cxcl1,* **(F)** *Ccl2,*** (G)** *Tnfa*, **(H)** *Il6* and **(I)** *Amy2* in the pancreas. **(J)** Western blot (top) and densitometry analysis (bottom) for cleaved caspase 3 levels in primary wild-type or *Lrg1^-/-^* acinar cells subjected to saline or caerulein treatment. qRT-PCR analysis of mRNA levels of **(K)** *Tnfa*, **(L)** *Il6,* and **(M)** *Cxcl1* in primary acinar cells isolated from wild-type or *Lrg1^-/-^* mice subjected to saline or caerulein treatment. All analyses were performed 24 hours after the induction of AP. Images are representative. Data are presented as the mean ± s.e.m. Significance was determined by one-way ANOVA followed by Holm-Sidak's multiple comparisons test of n ≥ 4 mice; *: p < 0.05, **: p < 0.01, ***: p < 0.001.

As demonstrated in **Figure 2**, multiple cell types contributed to elevated pancreatic LRG1 levels in AP. Myeloid cell-derived LRG1 has been previously implicated in diabetic wound healing 42 and cardiac remodelling 43. To explore the cellular source and the role of LRG1 in AP pathogenesis, wild-type and *Lrg1^-/^*^-^ mice were irradiated and transplanted with either wild-type or *Lrg1^-/^*^-^ BMCs before being subjected to caerulein-induced AP (**Figure 5A**). Unlike the wound healing process in other organs, H&E staining (**Figure 5B**) and histopathological scores (**Figure 5C**) revealed worse pancreatic injury in *Lrg1^-/-^* mice compared to those on a wild-type background, regardless of the BMC source. In addition, wild-type BMCs did not reduce the increased infiltration of MPO^+^ myeloid cells in the pancreas of *Lrg1^-/-^* mice (**Figure 5D**) potentially due to the elevated pancreatic levels of neutrophil chemoattractant *Cxcl1* (**Figure 5E**) and monocyte chemoattractant *Ccl2* (**Figure 5F**). Consistently, the expression of inflammatory cytokines, including *Tnfa* (**Figure 5G**), *Il6* (**Figure 5H**), was significantly higher in *Lrg1^-/-^* recipient mice than in wild-type controls, irrespective of the BMC source. A moderate increase in pancreatic IL-1β levels was also observed in *Lrg1^-/-^* recipients although it did not reach statistical significance (**Supplementary Figure S5A**). Additionally, these mice showed a concomitant decrease in pancreatic *Amy2* mRNA levels (**Figure 5I**). The ability of myeloid cells to adhere to the endothelium is central to the inflammatory process in different organs 44. LRG1's role in myeloid cell function was further validated using siRNA-mediated *Lrg1* knockdown (**Supplementary Figure S5B**). Our study showed that *Lrg1* knockdown in HL-60 promyelocytes did not affect their ability to adhere to human pancreatic microvascular endothelial cell (HPaMEC) monolayers in the presence or absence of TNFα (**Supplementary Figure S5C**), suggesting that LRG1 is dispensable for myeloid cell function in AP.

Given that pancreatic acinar cells were a major source of LRG1 and *Lrg1* deletion led to a marked acinar cell loss in AP, we further investigated *Lrg1*'s role in acinar cell function and signalling. Western blot analysis showed a significant increase in cleaved caspase 3 levels, indicating substantial acinar cell apoptosis 45 in primary acinar cells isolated from *Lrg1^-/-^* mice subjected to caerulein-induced AP (**Figure 5J**). In line with this observation, *Lrg1^-/-^* acinar cells expressed elevated levels of inflammatory cytokines and chemokines, including *Tnfa* (**Figure 5K**), *Il6* (**Figure 5L**), and *Cxcl1* (**Figure 5M**), compared to wild-type counterparts.

Calcium-mediated PKC, JAK/STAT, and JNK signalling pathways are crucial for mediating caerulein-induced proinflammatory responses in acinar cells 41. Indeed, primary acinar cells from *Lrg1^-/-^* mice subjected to AP induction showed a marked increase in phosphorylated PKCnu (pPKCnu), STAT3 (pSTAT3), and JNK (pJNK) levels (**Supplementary Figure S5D**). Together, these data suggest that Lrg1 deletion makes acinar cells more susceptible to caerulein-induced damage.

### Deletion of *Lrg1* enhances AP recovery

**Figure 6:**
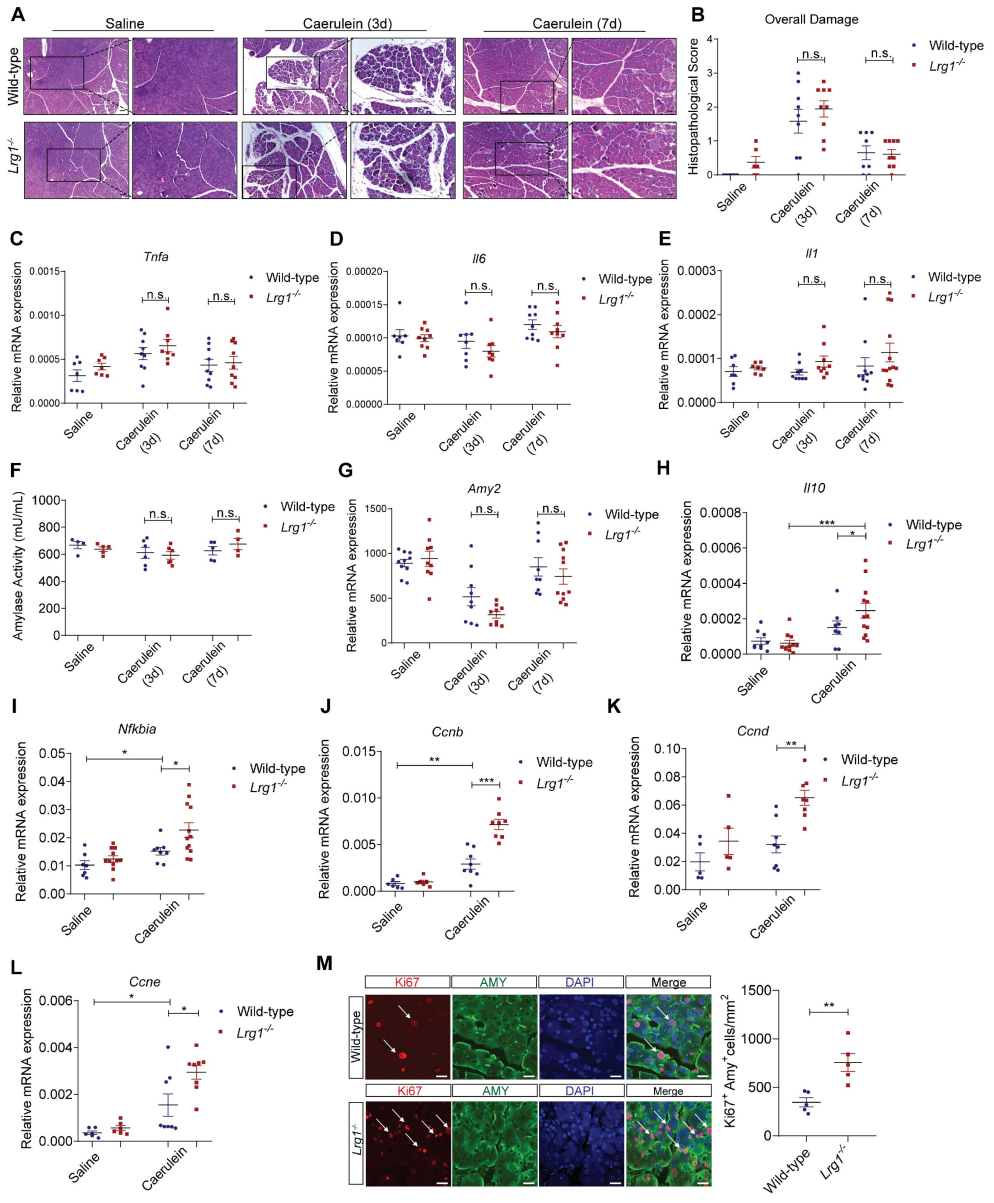
***Lrg1*-deletion benefits AP recovery in mice in AP. (A)** H&E staining and **(B)** histopathological grading demonstrating overall pancreatic damage. Scale bar: 50μm, Scale bar of boxed regions: 25μm. qRT-PCR analysis of mRNA levels of **(C)** *Tnfa*, **(D)** *Il6,* and **(E)** *Il1*. **(F)** Serum amylase activity in saline or caerulein-treated wild-type and *Lrg1*-deficient mice. qRT-PCR analysis of **(G)** *Amy2* mRNA levels in the pancreas. qRT-PCR analysis of mRNA levels of anti-inflammatory cytokines **(H)** *Il10* **(I)** *Nfkbia* and proliferative markers **(J)** Cyclin B, *Ccnb*, **(K)** Cyclin D, *Ccnd,* and **(L)** Cyclin E, *Ccne* in the pancreas. **(M)** Immunofluorescent staining against Ki67 (red), AMY (green), and DAPI (blue) (left) and quantification (right) of Ki67^+^ proliferating acinar cells (arrow) in the pancreas. Scale bar: 20μm. All images are representative. Figures **(A)**-**(G)** were performed in the pancreas of saline or caerulein-treated wild-type and *Lrg1*-deficient mice at 3- and 7-day post the induction of AP. Figures **(H)**-**(I)** and **(J)**-**(M)** were performed in the pancreas of saline- or caerulein-injected wild-type and *Lrg1^-/-^* mice 24 hours and 3 days post the induction of AP respectively. Data are presented as the mean ± s.e.m. Significance was determined by one-way ANOVA followed by Holm-Sidak's multiple comparisons test or unpaired, two-tailed Student's t-test of n ≥ 4 mice; *: p < 0.05, **: p < 0.01, ***: p < 0.001, n.s.: not significant, p > 0.05.

Although the exocrine pancreas remained severely damaged on day 3, histopathological analysis showed no obvious difference in overall pancreas structure between *Lrg1^-/-^* and wild-type mice (**Figures 6A and 6B**). By day 7, caerulein-induced AP had fully resolved in both genotypes (**Figures 6A and 6B**). Supporting these observations, the expression levels of inflammatory cytokines, such as *Tnfa* (**Figure 6C**), *Il6* (**Figure 6D**), and *Il1β* (**Figure 6E**) were comparable between wild-type and *Lrg1^-/-^* mice at both 3 and 7 days following caerulein treatment.

Consistently, pancreatic exocrine function as assessed by amylase activity (**Figure 6F**) and *Amy2* gene expression (**Figure 6G**), showed no difference between wild-type and *Lrg1^-/-^* mice during recovery. As observed in various preclinical models of AP, the resolution of inflammation and pancreatic regeneration is primarily achieved through the concurrent production of anti-inflammatory cytokines during the peak of inflammation 46, 47 followed by the expansion of surviving acinar cells in the resolution phase 48-50. Indeed, *Lrg1^-/-^* mice exhibited significantly higher levels of anti-inflammatory molecules *Il10* (**Figure 6H**) and *Nfkbia* (**Figure 6I**) 24 hours post-AP. Additionally, these mice also displayed an increased expression of cyclin genes essential for mitosis, including *Ccnb*, *Ccnd,* and *Ccne* (**Figure 6J-L**) on day 3 post-caerulein treatment which was further confirmed by immunofluorescence staining which demonstrated an increased number of proliferating Ki67^+^/AMY^+^ acinar cells in *Lrg1^-/-^* pancreas as compared to wild-type controls (**Figure 6M**).

Taken together, our data showed that *Lrg1* deletion accelerates AP recovery by promoting acinar cell proliferation.

### LRG1 regulates CCK1R expression *via* ALK5/AKT signalling in acinar cells

**Figure 7:**
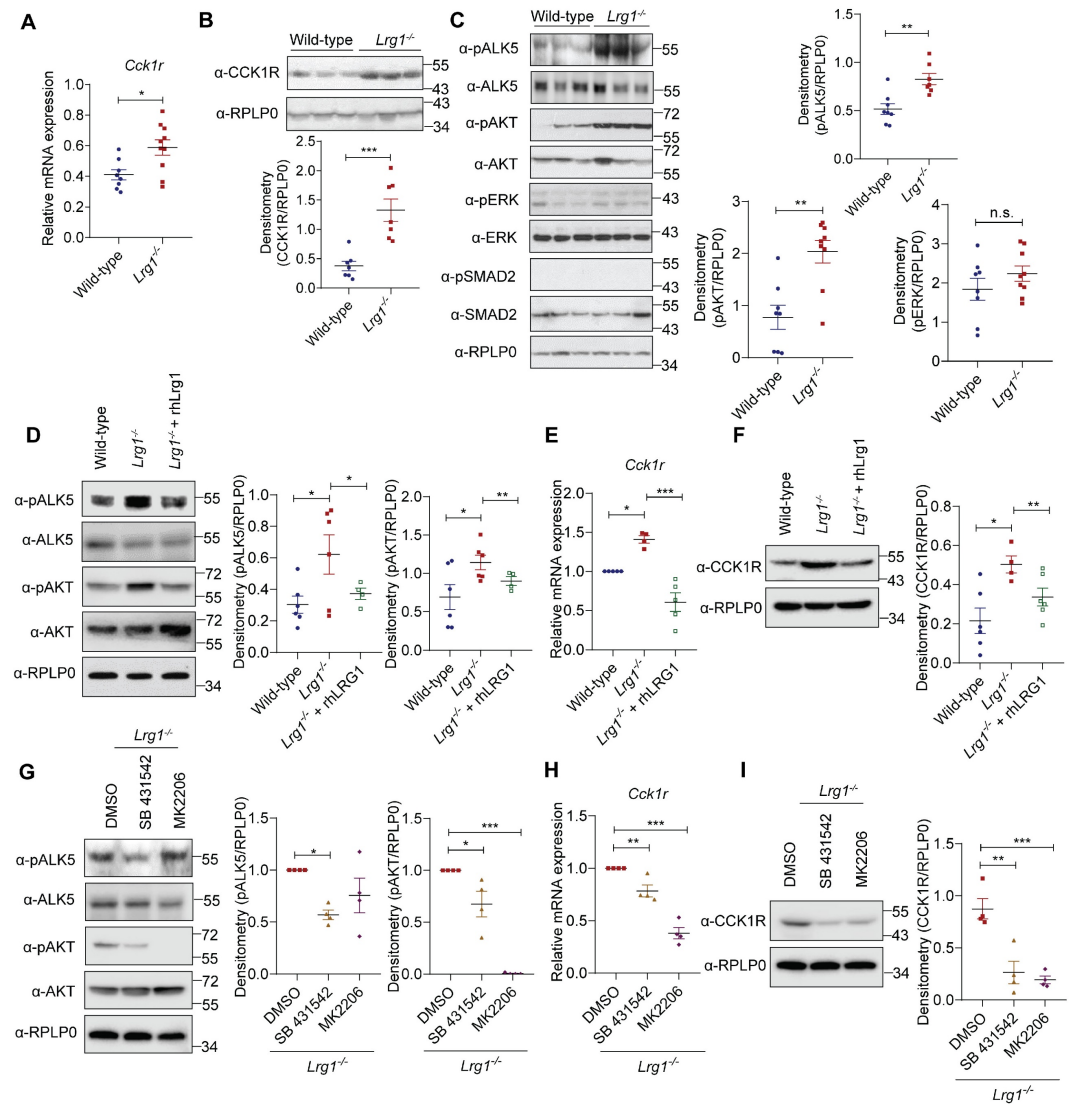
** LRG1 regulates acinar cell function through AKT-mediated CCK1R expression. (A)** qRT-PCR analysis of cholecystokinin A receptor (*Cck1r*) mRNA levels and **(B)** Western blot (top) and densitometry analysis (bottom) of CCK1R protein levels in the pancreas of adult wild-type and *Lrg1^-/-^* mice. **(C)** Western blot (left) and densitometry analysis (right) of phosphorylated and total levels of ALK5, AKT, ERK, and SMAD2 in the pancreatic of wild-type and *Lrg1*-deficient mice. **(D)** Western blot (left) and densitometry analysis (right) of phosphorylated and total levels of ALK5 and AKT protein in primary acinar cells isolated from wild-type or *Lrg1^-/-^* mice in the presence or absence of rhLRG1. **(E)** qRT-PCR analysis C*ck1r* mRNA levels and **(F)** Western blot (left) and densitometry analysis (right) of CCK1R protein levels in primary acinar cells isolated from wild-type or *Lrg1^-/-^* mice in the presence or absence of rhLRG1. **(G)** Western blot (left) and densitometry analysis (right) of pALK5, ALK5, pAKT, and AKT levels in primary acinar cells isolated from *Lrg1^-/-^* mice subjected to the treatment with ALK5 (SB 431542) or AKT(MK2206) specific inhibitor. **(H)** qRT-PCR analysis of C*ck1r* mRNA levels and **(I)** Western blot (left) and densitometry analysis (right) of CCK1R protein levels in primary acinar cells isolated from *Lrg1^-/-^* mice subjected to the treatment with ALK5 or AKT inhibitor. All images are representative. Data are presented as the mean ± s.e.m. Significance was determined by one-way ANOVA followed by Holm-Sidak's multiple comparisons test or unpaired, two-tailed Student's t-test of n ≥ 4 mice or independent experiments; *: p < 0.05, **: p < 0.01, ***: p < 0.001.

Caerulein activates both CCK1R and CCK2R 51. To investigate how LRG1 regulates AP development, we first analyzed the expression of CCK1R and CCK2R in the pancreas of *Lrg1^-/-^* and wild-type mice. In contrast to CCK1R (Ct ~ 18), CCK2R is expressed at a very low level (Ct ~ 30) in mouse pancreas as determined by quantitative RT-PCR (**Supplementary Figure S6A**). Interestingly, both mRNA (**Figure 7A**) and protein (**Figure 7B**) levels of CCK1R were significantly higher in the pancreas of *Lrg1^-/-^* mice, whereas CCK2R expression remain unaffected by *Lrg1* deletion (**Supplementary Figure S6B**). LRG1 is known to regulate both canonical and noncanonical TGFβ signalling in non-pancreatic cells 20, 42, 52. Next, we examined the activation of key TGFβ signalling transducers in *Lrg1^-/-^* and wild-type pancreas. While levels of phosphorylated ERK (pERK) and phosphorylated SMAD2 (pSMAD2) were unaffected, levels of phosphorylated type I TGFβ receptor ALK5 (pALK5) and phosphorylated AKT (pAKT) were significantly higher in *Lrg1^-/-^* mice (**Figure 7C**). Importantly, supplementation of recombinant human LRG1 (rhLRG1) significantly attenuated pALK5 and pAKT levels (**Figure 7D**), along with *Cck1r* mRNA (**Figure 7E**) and protein levels (**Figure 7F**) in *Lrg1^-/-^* acinar cells. Similarly, specific small molecule inhibitors of ALK5 (SB431542) or AKT (MK2206) significantly attenuated pALK5 and pAKT (**Figure 7G**) and CCK1R mRNA and protein (**Figures 7H and I**) levels in *Lrg1^-/-^* acinar cells. Given that CCK1R mediates caerulein-induced acinar cell damage, this TGFβ/ALK5/AKT dependent elevation in CCK1R may explain the more severe initial pancreatic injury observed in *Lrg1^-/-^* mice during caerulein-induced AP.

### CCK1R inhibition ameliorates AP severity in *Lrg1^-/-^* mice

**Figure 8:**
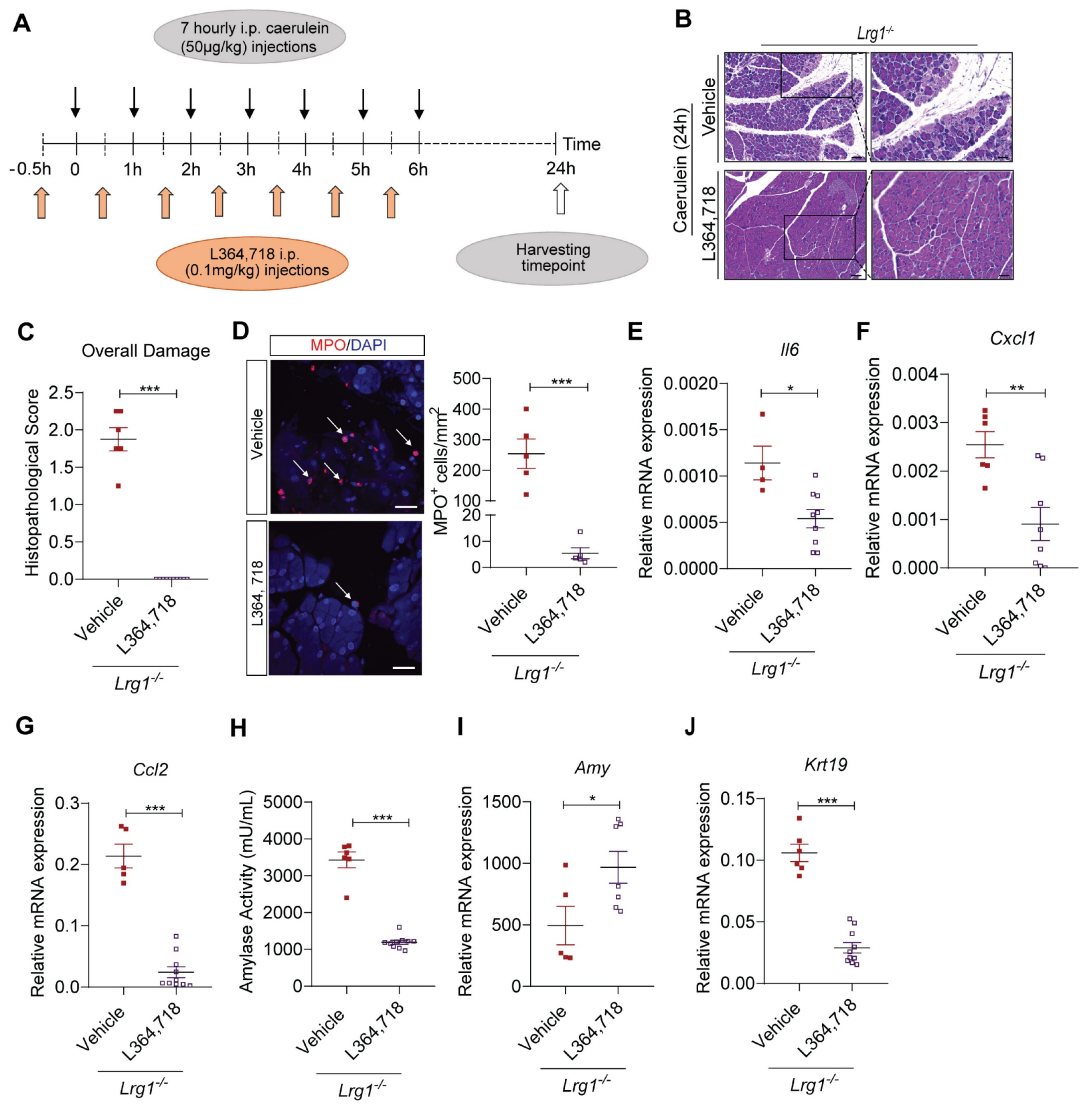
** LRG1 regulates AP pathology in a CCK1R-dependent manner. (A)** Schematic diagram of CCK1R antagonist L364,718 administration strategy. **(B)** H&E staining and **(C)** histopathological scoring of overall pancreatic damage in vehicle or L364,718-treated *Lrg1^-/-^* mice 24 hours after the induction of AP. Scale bar: 100μm, scale bar of boxed regions: 50μm. **(D)** Immunofluorescent staining against MPO (red) and DAPI (blue) (left) and quantification (right) of infiltrated MPO^+^ inflammatory cells (arrow) of the pancreas tissues of caerulein-treated *Lrg1^-/-^* mice following the treatment with either vehicle control or L364,718. Scale bar: 25μm. qRT-PCR analysis of pancreatic mRNA levels of **(E)** *Il6* and **(F)** *Cxcl1* **(G)** *Ccl2* in vehicle or L364,718 treated *Lrg1*-deficient mice following the induction of AP. **(H)** Serum amylase activity of the vehicle or L364,718 treated *Lrg1^-/-^* mice 24 hours following the AP induction. qRT-PCR analysis of pancreatic mRNA levels of **(I)** *Amy2* and **(J)** *Krt19*. All images are representative. Data are presented as the mean ± s.e.m. Significance was determined by unpaired, two-tailed Student's t-test of n ≥ 5 mice; *: p < 0.05, **: p < 0.01, ***: p < 0.001.

To further understand the role of CCK1R in early pancreatic damage in *Lrg1*^-/-^ mice, we treated *Lrg1^-/-^* mice with CCK1R inhibitor L364,718 30 minutes before caerulein injection (**Figure 8A**). Histopathological analysis performed 24 hours later revealed that L364,718 significantly attenuated the severity of AP in *Lrg1^-/-^* mice, as evidenced by decreased acinar cell loss, inflammatory cell infiltration, and interstitial edema based on H&E staining (**Figure 8B**) and histopathological scoring (**Figure 8C**). Immunofluorescence staining also showed that L364,718 strongly inhibited the infiltration of MPO^+^ cells into the pancreas of *Lrg1^-/-^* mice following caerulein treatment (**Figure 8D**). To support this observation, the expression of inflammatory cytokines, including *Il6* (**Figure 8E**), *Cxcl1* (**Figure 8F**), and Ccl2 (**Figure 8G**), was also significantly reduced in the pancreas of *Lrg1^-/-^* mice treated with L364,718, although no significant differences were observed in *Tnfa* (**Supplementary Figure S7A**) and *Il1β* (**Supplementary Figure S7B**) levels between vehicle and L364,718-treated mice. Improvement in pancreatic function was further confirmed by a marked reduction in circulating amylase activity (**Figure 8H**), increased pancreatic *Amy2* levels (**Figure 8I**) and decreased pancreatic *Krt19* expression (**Figure 8J**) in L364,718-treated *Lrg1^-/-^* mice compared to vehicle-treated controls. In addition, key signalling molecules associated with pancreatic injury, including pPKC, pSTAT3 and pJNK, were attenuated by L364,718 (**Supplementary Figure S7C**). These data reinforce the critical role of CCK1R in LRG1-mediated AP pathogenesis.

### LRG1 inhibition promotes AP recovery *in vivo*

**Figure 9:**
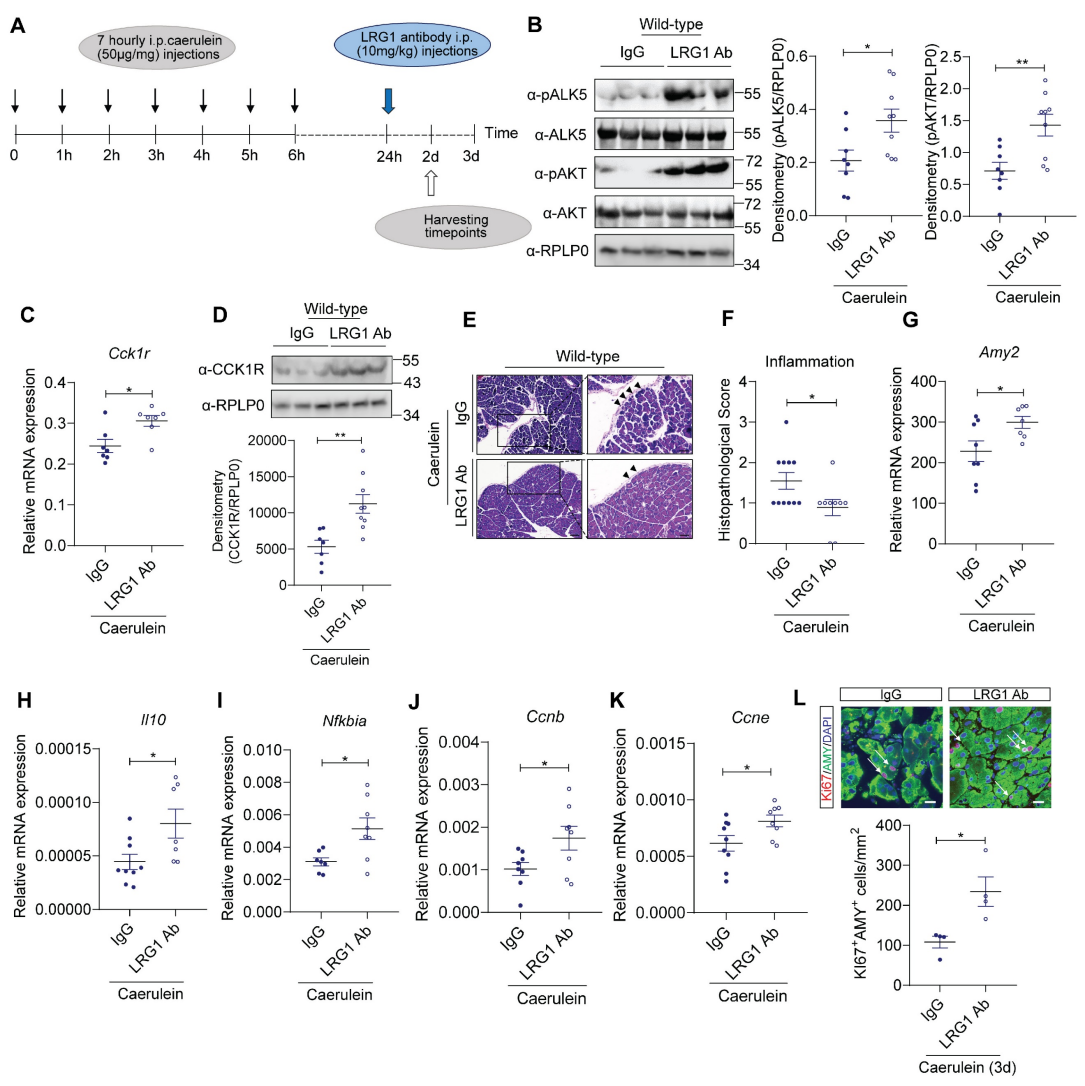
** LRG1 inhibition promotes pancreatic recovery in AP. (A)** Schematic diagram of LRG1 antibody treatment strategy. **(B)** Western blot (left) and densitometry analysis (right) of pancreatic phosphorylated and total ALK5 and AKT levels in AP mice treated with IgG or LRG1 antibody. **(C)** qRT-PCR analysis of pancreatic *Cck1r* mRNA levels in AP mice treated with IgG or LRG1 antibody. **(D)** Western blot (top) and densitometry analysis (bottom) of pancreatic CCK1R protein levels in AP mice treated with IgG or LRG1 antibody. **(E)** H&E staining demonstrating inflammatory cell infiltration (arrowhead) and **(F)** histopathological grading of the pancreas of AP mice subjected to IgG or LRG1 antibody treatment. Scale bar: 100μm, Scale bar of boxed regions: 50μm. qRT-PCR analysis of pancreatic **(G)** *Amy2*, **(H)** *Il10*, **(I)** *Nfkbia,* and **(J)** *Ccnb* **(K)** *Ccne* levels in IgG or LRG1 antibody-treated mice at Day 2 following AP induction. **(L)** Immunofluorescent staining against Ki67 (red), AMY (green), and DAPI (blue) (top) and quantification (bottom) of Ki67^+^ acinar cells (arrow) in IgG and LRG1 antibody-treated mice at Day 3 post-AP induction. Scale bar: 20μm. Data are presented as the mean ± s.e.m. All images are representative and all experiments are conducted 2 days post-AP induction unless specified. Significance was determined by unpaired, two-tailed Student's t-test of n ≥ 5 mice; *: p < 0.05, **: p < 0.01.

Despite the more severe initial pancreatic injury in *Lrg1^-/-^* mice, there is no difference, in terms of pancreatic structure and function, between *Lrg1^-/-^* and wild-type mice at day 3 and day 7 post-caerulein treatment, suggesting *Lrg1* deletion benefits AP recovery. To test this hypothesis, mice were treated with monoclonal neutralizing LRG1 antibody or control IgG 24 hours after the first caerulein administration, coinciding with the peak of LRG1 expression, as illustrated in **Figure 9A**. As expected, LRG1 neutralizing antibody significantly increased pALK5, pAKT levels (**Figure 9B**), and CCK1R at both mRNA (**Figure 9C**) and protein levels (**Figure 9D**) in AP mice. The increase in pALK5, pAKT and CCKAR (**Supplementary Figure S8A and S8B**) was also recapitulated in primary murine acinar cells following LRG1 antibody treatment. Importantly, in AP mice, LRG1 antibody improved pancreatic structure and function, as demonstrated by H&E staining (**Figure 9E**), histopathological scores for overall inflammation (**Figure 9F**), and pancreatic *Amy2* mRNA levels (**Figure 9G**). Furthermore, LRG1 neutralizing antibody markedly promoted the expression of anti-inflammatory molecules, *Il10* (**Figure 9H**) and *Nfkbia* (**Figure 9I**).

Using a caerulein-independent PDL model of AP, administration of LRG1 neutralizing antibody induced CCK1R expression (**Supplementary Figure S8C**), improved overall pancreatic structure and function, as evidenced by H&E staining (**Supplementary Figure S8D**), reduced histopathological scores for overall damage (**Supplementary Figure S8E**) and increased pancreatic *Amy2* mRNA levels (**Supplementary Figure S8F**).

Besides its role in caerulein-induced AP, CCK1R also mediates trophic and proliferative effects of CCK in the pancreas 53-55. Our data showed that the expression of cell proliferation markers, such as *Ccnb* (**Figure 9J**) and *Ccne* (**Figure 9K**), as well as the number of Ki67^+^ acinar cells, were significantly increased in LRG1 antibody-treated caerulein-induced AP mice (**Figure 9L**).

Importantly, the administration of LRG1 antibody in wild-type mice did not trigger overt toxicity or side effects as seen from normal white blood cell (WBC) counts (**Supplementary Figure S9A**) and H&E-stained images of key organs such as liver, kidney, and heart (**Supplementary Figure S9B**) compared to IgG controls. Taken together, these data suggest the safety and efficacy of targeting Lrg1 with a neutralizing antibody to promote AP recovery by stimulating acinar cell proliferation and reducing pancreatic inflammation.

## Discussion

AP is a complex and dynamic disease involving multiple types of cells, molecular modulators, and signalling pathways, which makes it challenging to develop targeted therapeutics. While recent advances shed light on the roles of antioxidants, anticoagulants, proteinase inhibitors, autophagy inhibitors, etc. in reducing disease severity 56, clinical evidence supporting their use is limited. Irrespective of the causes, pancreatic acinar cell damage is central to AP pathophysiology. Strategies to stimulate acinar cell regeneration could be promising in mitigating pancreatic injury and preventing the development of multiorgan dysfunction syndrome and mortality in AP.

LRG1 is a multifunctional glycoprotein that was previously reported to regulate cell mitosis 22, 31, angiogenesis 20, and inflammatory response 31, 57. As *Lrg1^-/-^* mice are viable and fertile without demonstrating overt phenotypic abnormalities, LRG1's physiological roles remain poorly defined. Similar to what has been reported in other healthy tissues, Lrg1 is predominantly expressed in the vasculature of mouse and human pancreas. However, *Lrg1* deletion caused no changes in vessel density, nor did it affect the structural integrity or functional capacity of the exocrine and endocrine pancreas in mice. These observations suggest the existence of a potential compensatory mechanism for LRG1. Tsukushi (TSK), LRRC32 and LRRC33 are leucine-rich repeat proteins structurally related to LRG1 58. Similar to LRG1, they have been reported to regulate TGFβ1 activation and signalling in different types of cells 59, 60. Importantly, all three leucine-rich repeat proteins are dominantly expressed in the exocrine pancreas 61, 62. It will be interesting to see whether these proteins share overlapping roles with LRG1 in the pancreas under physiological conditions.

Unlike its physiological role, LRG1 has been extensively studied under pathological conditions. LRG1 was first reported as an important regulator of pathological neovascularization in the eye 20. It was then linked to various fibrovascular complications, including cancers, diabetic vascular complications, cardiovascular diseases, neurodegenerative disorders, and inflammatory diseases 31. Here we observed a significant elevation in serum LRG1 levels in human patients with AP, consistent with its reported role as an acute-phase protein 17. Importantly, our data demonstrated a strong correlation between serum LRG1 levels and established biomarkers of AP severity, such as CRP concentration, serum lipase, and blood neutrophil levels, underscoring its potential as an adjunctive tool for AP diagnosis and prognosis. Moreover, as an acute-phase protein, LRG1 may offer insights into ongoing inflammation and tissue damage, potentially guiding therapeutic interventions and monitoring treatment efficacy. Further studies are needed to evaluate the sensitivity and specificity of LRG1 as a comprehensive diagnostic and prognostic tool, potentially improving patient stratification, treatment decisions, and clinical outcomes in AP.

Bone marrow-derived myeloid cells serve as a major source of LRG1 and play critical roles in cardiac and dermal wound-healing processes 42, 43. Myeloid cells also play a critical immune modulation role in AP 14. Following their activation by the initial acinar cell damage, they are recruited to the injury site and further amplify the inflammatory signals. Although myeloid cells express LRG1 following the induction of AP, they are dispensable for LRG1-mediated AP pathogenesis as irradiated *Lrg1^-/-^* mice demonstrated a worse AP phenotype than wild-type counterparts irrespective of the source of donor bone marrow. Besides myeloid cells, we noted that LRG1 was strongly induced in pancreatic acinar cells in both human AP patients and mice subjected to caerulein-induced or PDL-induced AP. To understand the regulatory control of LRG1 in acinar cells, we performed *in silico* analysis of the LRG1 promoter and identified two putative binding sites of STAT3. STAT3 is a transcription factor that controls a variety of cellular processes, including cell survival, proliferation, differentiation, and inflammation 41. In the context of AP, STAT3 activation serves to amplify inflammatory response by promoting the transcription of pro-inflammatory cytokines, thus creating a positive feedback loop that sustains the inflammatory milieu. This process is critical for timely clearance of damaged acinar cells and pancreatic tissue regeneration 41. Indeed, mice with pancreatic-specific deletion of STAT3 demonstrated increased serum amylase and worse pancreatic pathologies 63, which were similar to what we observed in *Lrg1^-/-^* mice. On the other hand, abnormal pancreatic STAT3 activation contributes to dysregulated extracellular matrix remodelling 64, the development of post-endoscopic retrograde cholangiopancreatography (ERCP) pancreatitis 65 and secondary complications of severe AP 66. STAT3 is a common signalling transducer of interleukin (IL) 6 superfamily of cytokines 41. IL-6 is one of the first inflammatory mediators released in AP and elevated IL-6 serves as an early predictive biomarker for severe AP 38, 67. Following the recruitment of either transmembrane receptor mIL-6R or alternative spliced soluble IL-6 receptor (sIL-6R), and ubiquitously expressed gp130 protein, IL-6 induces the phosphorylation of STAT3 in acinar cells 66, 68. Consistent with the important role of STAT3 in AP, the deletion of IL-6 in mice leads to increased inflammatory response and worse pancreatic pathology following the caerulein-induced AP 69. Intriguingly, serum LRG1 and IL-6 levels are positively correlated in AP patients and both pancreatic and serum Lrg1 level mirrors that of IL-6 in mice subjected to caerulein-induced AP. We further showed that IL-6 induced LRG1 expression in pancreatic acinar cells and the IL-6-induced pancreatic LRG1 expression could be completely attenuated by STAT3 inhibitor Stattic. These data provide compelling evidence that LRG1 serves as a direct target gene of IL-6/STAT3 in pancreatic acinar cells and may mediate their action in AP pathogenesis.

Amongst the experimental murine models of AP, pancreatic secretagogue-induced pancreatitis is a highly reproducible model that relies on the administration of supraphysiological doses of cholinergic agonists like CCK or its analogue, caerulein to induce pancreatic damage 36. CCK exerts its function through two distinct transmembrane G-protein-coupled receptors, cholecystokinin type 1 receptor (CCK1R) and cholecystokinin type 2 receptor (CCK2R) 51. Our study showed that CCK1R but not CCK2R is expressed in the mouse pancreas, and CCK1R levels are significantly higher in the pancreas of *Lrg1^-/-^* mice, which may contribute to the exacerbated AP observed in these mice. The role of CCK1R in LRG1-mediated AP pathology was further supported by a significant attenuation of pancreatic injury in *Lrg1^-/-^* mice following the treatment with CCK1R inhibitor L364,718.

Several studies reported the ability of the adult pancreas to regenerate after the injury 15, 16 and surviving acinar cells can dedifferentiate and proliferate 15. Besides its important role in pancreatic enzyme secretion, CCK promotes DNA synthesis, nuclear labelling, the total content of DNA and protein 70, and the regeneration of the exocrine pancreas 71-73. In support of CCK1R's role in the trophic action of CCK, treatment with a CCK1R-specific agonist leads to increased pancreas weight in non-human primates 74. CCK1R is also highly enriched in neoplastic human tissues 75 and pancreatic cancers 76. Consistent with elevated CCK1R levels in the pancreas of* Lrg1^-/-^* mice, our study showed that the percentage of proliferating acinar cells and the expression levels of cell cycle components were significantly higher in the pancreas of *Lrg1^-/-^* mice compared to the wild-type controls following caerulein-induced AP. It is worth noting that the accelerated recovery of pancreatic injury in *Lrg1^-/-^* mice could also be attributed to elevated expression of key anti-inflammatory cytokines, *Il10* and *Nfkbia*. Indeed, as a mechanism to restore homeostasis, the peak stage of inflammation in AP is usually accompanied by compensatory CARS to promote resolution of inflammation and tissue repair 11, 12.

**Figure 10:**
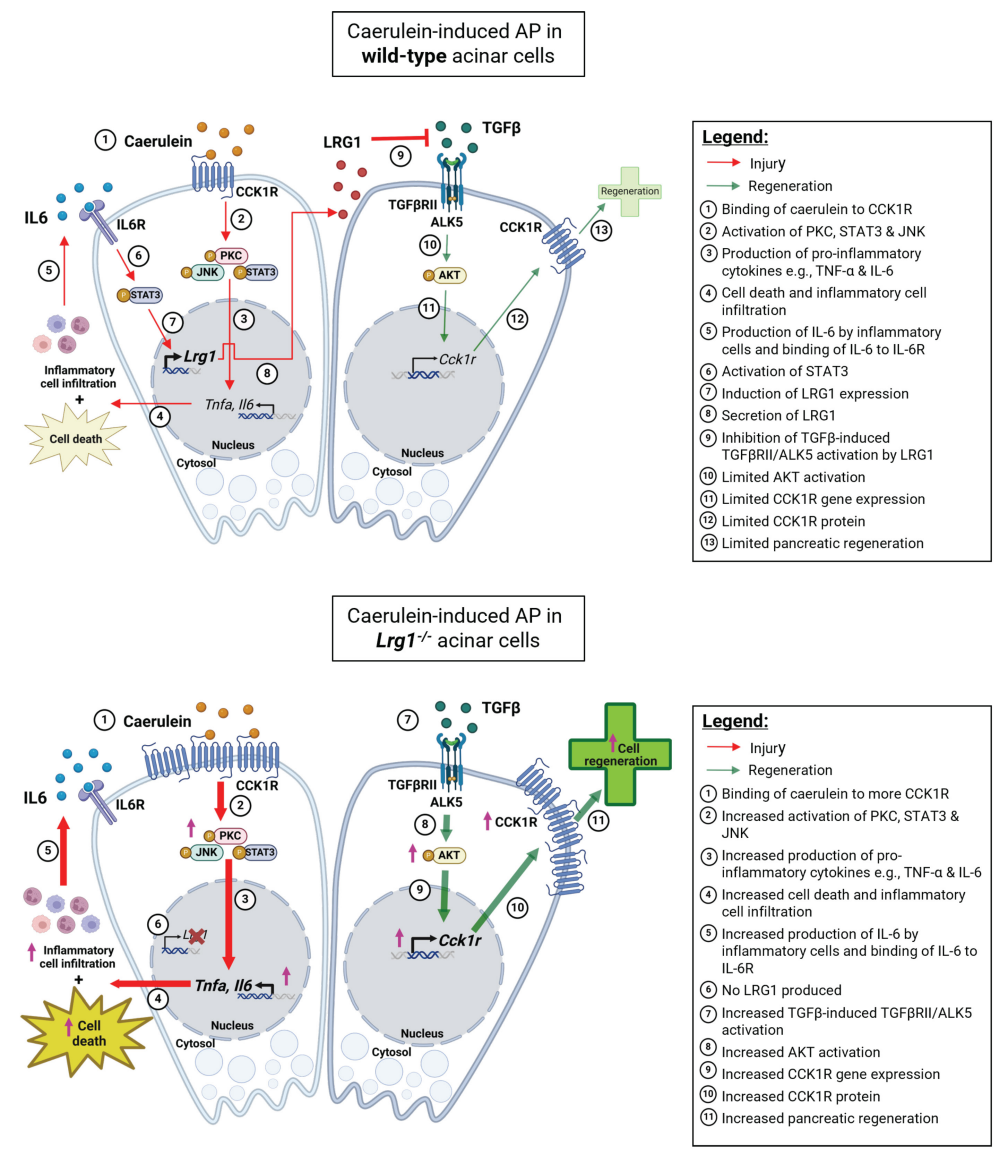
** Mechanistic summary of the role of LRG1 in pancreatic injury and regeneration.** In wild-type acinar cells, the binding of caerulein to CCK1R on the surface of acinar cells triggers a cascade of signaling events, including the activation of PKC, STAT3, and JNK pathways. This leads to acinar cell apoptosis and the production of pro-inflammatory cytokines such as TNF-α and IL-6, which amplify the inflammatory response initiated by the initial acinar cell injury. Concurrently, IL-6 signals through its receptor, IL-6R, to activate the transcription factor STAT3, which subsequently induces LRG1 expression in acinar cells. LRG1, in turn, antagonizes the TGFβRII/ALK5/AKT-mediated expression of CCK1R, a trophic factor for acinar cells, thereby limiting pancreatic regeneration. In *Lrg1^-/-^* acinar cells, the inhibitory effect of LRG1 on the TGFβRII/ALK5/AKT pathway is absent, resulting in elevated CCK1R expression compared to wild-type acinar cells. Consequently, more CCK1R is available to bind caerulein, leading to greater acinar cell damage. However, this higher CCK1R expression also promotes increased acinar cell proliferation and regeneration, explaining the accelerated pancreatic regeneration observed in *Lrg1^-/-^* mice despite the presence of more severe initial damage.

*Lrg1* was previously reported to exert its function through regulating TGFβ signalling in different disease contexts 31. To understand the molecular mechanisms of LRG1-mediated pancreatic injury and recovery in caerulein-induced AP, we studied the activation of canonical and non-canonical TGFβ signalling transducers in the pancreas and showed that *Lrg1* deletion or supplementation specifically affects the ALK5-mediated AKT activation. Intriguingly, the elevated CCK1R expression in *Lrg1* knockout acinar cells was significantly suppressed by an ALK5 or AKT inhibitor. These data suggest that the overactivation of noncanonical TGFβ/ALK5/AKT signalling and the subsequent up-regulation in CCK1R, not only makes *Lrg1^-/-^* mice more susceptible to caerulein-induced acinar cell injury but also explains the increased pancreatic regeneration following the AP in the absence of *Lrg1*. These dynamics are further summarized in **Figure 10**.

While this study provided evidence for the involvement of CCK1R in LRG1-mediated pancreatic exocrine regeneration, existing literature suggests that the process of transient acinar-ductal metaplasia, proliferation and re-differentiation 15 also engages key regenerative pathways, such as Notch, Wnt/β-catenin and Hedgehog signaling 48, 49, 77. As LRG1 has previously been implicated in these pathways 78, 79, it would be interesting to investigate whether these pathways converge or interact in the context of tissue regeneration in the pancreas and other organs.

It is important to acknowledge the ongoing debate regarding the clinical relevance of CCK1R in human AP 76, 80, 81. However, recent landmark studies provide compelling evidence of CCK1R expression in the human pancreas 82. In addition, both human pancreatic acinar cells 83 and *ex-vivo* human pancreatic slices 84 have been shown to respond directly to CCK by inducing robust calcium signaling, rather than relying solely on indirect neural pathways. While cholinergic vagal innervation remains the dominant pathway for CCK-mediated stimulation of pancreatic function in humans, studies have also demonstrated that CCK1R activation through this pathway can exert inhibitory effects on inflammation and improve inflammatory disease outcomes 85, 86. This finding aligns with our observations of the beneficial effects of elevated CCK1R expression on pancreatic regeneration in *Lrg1^-/-^* mice. Nevertheless, an important avenue for future research would be to validate our key findings using human pancreatic tissues or organoids, which would further strengthen the translational relevance of our study.

Given that *Lrg1* deletion leads to increased expression of anti-inflammatory cytokines, CCK1R, cell cycle components, and acinar cell proliferation, blocking the action of LRG1 presents an attractive strategy to limit AP injury and boost the recovery. Indeed, LRG1 antibody protected the pancreas from both caerulein-dependent and caerulein-independent (pancreatic duct ligation induced) acinar cell injury and and promoted acinar cell regeneration. Demonstrating the protective role of LRG1 inhibition in both AP models is particularly significant, especially considering the inherent variability of murine AP models, including differences in pathophysiology, severity, duration, and reproducibility. This variability complicates the interpretation and clinical translation of findings. For example, while the caerulein model induces a mild form of AP, closely mimicking the molecular changes observed in acinar cells of human patients 36, the PDL model replicates gallstones-induced pancreatitis, a more severe form of AP, through a caerulein-independent mechanism 35. These model-specific differences highlight the challenge of achieving consistent results across experimental systems. However, by employing these complementary models, we consistently observed the therapeutic efficacy of the LRG1-blocking antibody, which strengthens the foundation for translating our findings into clinical applications. In fact, to further enhance the clinical utility of our work, it may also be worthwhile in future to investigate the role of LRG1-blocking antibody in other severe AP models known to induce extra-pancreatic damage (e.g., AP associated acute lung injury), such as pancreatic duct infusion of sodium taurocholate, choline-deficient diet enriched with ethionine 35 or the combination of caerulein and lipopolysaccharide 87.

Importantly, while preliminary data provides reassurance regarding the safety of the LRG1 antibody, the pleiotropic role of LRG1 in inflammation and tissue repair across various organs, along with its known interactions with multiple signaling pathways, warrants cautious consideration. To ensure the safety of LRG1 inhibition, it is crucial to further investigate potential side effects. In addition, comprehensive studies on dosing strategies and treatment regimens are necessary to maximize therapeutic efficacy while minimizing potential adverse effects. These studies should also take into account that despite significant conservation of function, interspecies differences in pharmacokinetics due to the cytochrome P450 system 88 and discrepancies in both the innate and adaptive immune system response to challenge in humans and mice 89 could potentially lead to varied drug metabolism and response to treatment and hence complicate interpretation of preclinical findings.

In summary, our study demonstrated the complex role of LRG1 in AP development and recovery. LRG1 inhibition by a neutralizing antibody is effective in promoting acinar cell regeneration and mitigating pancreatic damage, suggesting its promising role in preventing the progress to severe AP in clinical settings. The modulating effect of LRG1 on CCK1R expression is also significant, raising the possibility of LRG1's influence on other diseases in which CCK1R is known to play a role, such as metabolic 90, 91, neurological 92, 93 and other gastrointestinal disorders 94.

## Methods

### Sex as a biological variable

Our study examined both male and female animals and no sex differences were observed. In humans, the proportion of males and females with AP are also similar 95.

### Human pancreatitis specimens

Formalin-fixed, paraffin-embedded (PFFE) human pancreatitis tissues (#PA691) were obtained from TissueArray.com.

### Animals and AP induction

C57BL/6N mice were purchased from InVivos (Singapore). *Lrg1^-/-^* mice on a C57BL/6N background were purchased from the University of California, Davis, Knockout Mouse Project (KOMP) (http://www.komp.org). AP was induced in 10 to 12-week-old mice *via* 7 times hourly intraperitoneal injections of supramaximal concentrations of caerulein (50μg/kg, #HY-A0190, MedChemExpress, USA). Saline (0.9% NaCl) was used as a vehicle control. Pancreatic duct ligation (PDL) surgery was performed as previously described 96. The pancreas was visualized using a stereomicroscope and the tail region of the main pancreatic duct was ligated with 7-0 nonabsorbable, polypropylene suture (#M8703, Ethicon, USA). Mice were sacrificed at relevant time points by CO_2_ asphyxiation.

### CCK1R antagonist and LRG1 neutralizing antibody treatment in-vivo

L364, 718 (#HY-106301, MedChemExpress, USA), a competitive non-peptide antagonist of CCK1R was administered intraperitoneally in *Lrg1^-/-^* mice at a dose of 0.1mg/kg, 30 minutes before each caerulein injection. Mice that received the vehicle 10% DMSO dissolved in SBE-β-CD (#HY-17031, MedChemExpress, USA) served as controls. C57BL/6N mice were treated with LRG1 monoclonal antibody at the concentration of 10mg/kg intraperitoneally at 24 hours after the first caerulein treatment. Mice that received the corresponding isotype antibody (#BE0093, BioXCell, USA) served as controls.

### Bone marrow transplantation

10 to 12-week-old wild-type or *Lrg1^-/-^* mice were lethally irradiated at a fractionated dose of 5.5Gy, twice in a 4-hour interval using a BIOBEAM GM γ irradiation device (Gamma-Service Medical, Germany). Bone marrow cells were harvested from the bilateral tibia and femur bones of isogeneic donor mice and filtered through a 70μm cell strainer. 5x10^6^ bone marrow cells were delivered to irradiated recipient mice through the tail vein 24 hours after the irradiation. AP was induced in recipient mice 5 weeks post-transplantation as described above.

### Fasting blood glucose and insulin tolerance test (ITT)

Tail-tip blood samples were collected from mice that have undergone overnight fasting and tested for blood glucose measurement using point-of-care glucometer (Accu-Chek Performa, Switzerland). For ITT test, mice were fasted for 4 hours before being subjected to intraperitoneal injections of human insulin (0.75U/kg, #19278, Sigma-Aldrich, USA). Blood glucose levels were determined at 0, 15, 30, 60- and 120 minutes post-insulin injection.

### Histology and Immunofluorescence staining

Mouse pancreatic tissues were fixed in 4% paraformaldehyde (#158127, Sigma-Aldrich, USA) overnight before being embedded in paraffin or O.C.T compound following a standard protocol. Paraffin sections (6μm) were subjected to hematoxylin-eosin staining followed by histopathological grading by an independent pathologist (Advanced Molecular Pathology Laboratory, Institute of Molecular and Cell Biology, A*Star, Singapore). A score of 0 indicated that there were no abnormalities detected; 1: minimal; 2: mild; 3: moderate; 4: marked; 5: severe 97 (see **Supplementary Table S1**).

For immunofluorescence staining, paraffin (6μm) or cryosections (8µm) were subjected to antigen retrieval using sodium citrate buffer before being stained with primary antibodies against LRG1 (#13224-1-AP, Proteintech, USA), CD31 (#550274, BD Biosciences, USA or #ab28364, Abcam, UK), AN2 (#130-100-468, Miltenyi Biotec, Germany), Glucagon (#ab92517, Abcam, UK), Insulin (#ab ab7842, Abcam, UK), Amylase (#sc-46657, Santa-Cruz Biotechnology, USA), Cytokeratin 19 (#PAB12676, Abnova, USA), Myeloperoxidase (MPO) (#ab9535; Abcam, UK or #AF3667, R&D Systems, USA) and Ki67 (#ab15580; Abcam, UK) followed by staining with DAPI, Alexa 488, Alexa 594 and Alexa 657 secondary antibodies (Thermo Fisher Scientific, USA). Images were captured using Leica DM5500 microscope (Leica Microsystems, USA) or Carl Zeiss LSM 710 confocal microscopy (Zeiss, Germany) and analysed using Adobe Photoshop software (Adobe Inc, USA).

For vasculature quantification, the number of CD31+ vessels surrounding a unique nucleus was manually counted using the Count tool in Adobe Photoshop. Vessel density was then calculated by dividing the total number of CD31+ vessels by the imaged pancreatic area. A minimum of five random fields of view were analyzed per pancreas.

### Cells and cell culture

Mouse primary acinar cells were isolated using a standard collagenase IA (#C9891, Sigma-Aldrich, USA) digestion protocol as previously described 98. Human promyelocytic leukemia cell line, HL-60 (#CCL-240, ATCC, USA) and human pancreatic microvascular endothelial cells, HPaMEC (#3800, ScienCell Research Laboratories, USA) were maintained according to the supplier's instructions. Cells were treated with caerulein (1μM, #HY-A0190, MedChemExpress, USA), recombinant human IL-6 (100ng/mL, #200-06, PeproTech, USA), recombinant human LRG1 (200ng/mL, #7890-LR-025, R&D systems, USA), ALK5 inhibitor, SB431542 (10μM, Sigma-Aldrich, USA) and AKT inhibitor, MK-2206 (10μM, MedChemExpress, USA) as indicated.

### Quantitative RT-PCR

Pancreatic tissues were stored in RNAlater™ Stabilization Solution (#AM7020, Thermo Fisher Scientific, USA). Total RNA was isolated and purified from the mouse pancreas or cultured cells using RNAeasy kit (#74106, Qiagen, USA) and NucleoSpin RNA kit (#740955, Macherey-Nagel, Germany) respectively, before being converted to cDNA using qScript cDNA Supermix (#157031, Quanta Biosciences, USA). PCR was conducted with PrecisionFAST qPCR MasterMix (PPLUS-machine type-1ML, Primer Design, UK) using Applied Biosystems StepOnePlus™ Real-Time PCR System (Life Technologies, USA). The expression levels of respective target genes were normalized to RPLP0, and relative gene expressions were calculated using standard 2^-ΔΔCT^. PCR primer sequences are listed in **Supplementary Table S2**.

### SDS-PAGE and western blotting

Cells or tissues were lysed on ice in radioimmunoprecipitation assay buffer containing 1x protease inhibitor (1 tablet in 500uL, #04693116001, Roche, Switzerland), 1 mmol/L dithiothreitol (#10197777001, Sigma-Aldrich, USA), and 1 mmol/L phenylmethylsulfonyl fluoride (#P7626, Sigma-Aldrich, USA). Additional 100 mmol/L phosphatase inhibitors (#07575-51, Nacalai Tesque, Japan) were used when detecting cell signalling transducers. Proteins were separated by 10% SDS-PAGE before being transferred onto an Immobilon-PSQ PVDF Membrane (#IPVH00010, Merck Millipore, USA). Blots were probed with LRG1 antibody (rabbit monoclonal, #13224-1-AP, Proteintech,USA), phospho-PKC δ antibody (mouse monoclonal (#sc-365969, Santa-Cruz Biotechnology, USA), phospho-PKC epsilon antibody (rabbit polyclonal, #ab63387, Abcam, UK), PKC antibody (mouse monoclonal, #sc-17769, Santa-Cruz Biotechnology, USA), phospho-STAT3 (rabbit monoclonal, #9145, Cell Signaling Technology, USA), STAT3 (rabbit monoclonal, #12640, Cell Signaling Technology, USA), phospho-SAPK/JNK (rabbit monoclonal, #9255, Cell Signaling Technology, USA), SAPK/JNK (rabbit monoclonal, #9145, Cell Signaling Technology, USA), cleaved caspase 3 antibody (rabbit monoclonal, #9664, Cell Signaling Technology, USA), phospho-PKCnu antibody (rabbit polyclonal, #orb4440, Biorbyt, UK), PKCnu antibody (rabbit polyclonal, #bs-4157R, Bioss Inc, USA), CCK1R antibody (rabbit polyclonal, #bs-11514R, Bioss Inc, USA), phospho-Akt antibody (rabbit monoclonal, #4060; Cell Signaling Technology), Akt antibody (rabbit monoclonal, #9272; Cell Signaling Technology), phospho-p44/42 MAPK (ERK1/2) antibody (rabbit monoclonal, #4370, Cell Signaling Technology, USA), p44/42 MAPK (ERK1/2) antibody (rabbit monoclonal, #4695, Cell Signaling Technology, USA), phospho-Smad2 antibody (rabbit monoclonal, #3108, Cell Signaling Technology, USA), Smad2 antibody (rabbit monoclonal, #5339, Cell Signaling Technology, USA), HSP60 antibody (rabbit monoclonal, #12165, Cell Signaling Technology, USA), RPLP0 antibody (rabbit polyclonal, #11290-2-AP, Proteintech,USA), GAPDH antibody (mouse monoclonal, #sc-32233, Santa-Cruz Biotechnology, USA), followed by horseradish peroxidase-conjugated secondary antibodies (Bethyl Laboratories, USA). Densitometry was performed using ImageJ.

### Serum protein analyses

Mouse serum amylase activity and LRG1 were measured by an amylase activity assay kit (#MAK009, Sigma-Aldrich, USA) and a LRG1 ELISA kit (#27785 for mouse serum and #27769 for human serum, Immuno-Biological Laboratories, Japan), respectively, according to the manufacturer's instructions. For the fasting glucagon test, mice fasted overnight before blood collection for glucagon analysis by Glucagon EIA Kit (#EIA-GLU-1, RayBiotech, USA) following the manufacturer's instructions.

### Neutrophil adhesion assay

*Lrg1*-knockdown HL-60 cells were labelled with CellTracker™ Red CMTPX Dye (#C34552, Life Technologies, USA) before being seeded on a TNF-α (50ng/mL; #300-01A, PeproTech, USA)-treated confluent HPaMEC monolayer. Two hours later, nonadherent HL-60 cells were removed and adherent HL-60 cells were imaged using EVOS M5000 Imaging System (#AMF5000, Life Technologies, USA) and quantified using Adobe Photoshop software (Adobe Inc, USA).

### Molecular biological methods

siRNA oligonucleotides (#L-015179-01; Dharmacon, USA) were used for knocking down LRG1 with non-targeting siRNA (#D-001810-01; Dharmacon, USA) as a negative control. Transfection in dHL-60 cells was performed using RNAiMAX (#13778150, Thermo Fisher Scientific, USA) according to the manufacturer's protocol.

### Chromatin immunoprecipitation assay (ChIP)

Pancreatic acinar cells were cross-linked with 1% formaldehyde solution (#F8775, Sigma Aldrich, USA) and further processed using 130W ultrasonic processor unit connected to a 138 mm by 6 mm tip probe (#Z412619, Sigma Aldrich, USA). ChIP was performed using STAT3 (#12640, Cell Signaling Technology, USA) antibody with IgG (#2729, Cell Signaling Technology, USA) as a control following the manufacturer's instructions. Primers used to detect STAT3 bound LRG1 promoter at -63 to -72bp upstream from TSS are forward, 5′-GTAGAGCAATCCCCACCTCA-3′; reverse, 5′-CCGGGTCAACATTGCTACAT-3′, and forward, 5′-GCTTGGTTCCTTGTGGAAGAC-3′; reverse, 5′-GCAATAAGAGCAGGAACGTGG-3′ for -244 to -255bp upstream from TSS.

### Statistics

Data are presented as mean ± standard error of the mean (s.e.m.). Statistical analyses were performed by an unpaired, two-tailed Student's t-test, or one-way ANOVA followed by post-hoc Holm-Sidak's multiple comparisons test for datasets with multiple groups using Prism 8 (GraphPAD Software Inc.). Statistical details for each experiment, including n values, are provided in figure legends. All animal models employ adequately powered sample sizes as determined using statistical methods (D = 0.5, Zpwr = 1.282, Zcrit = 1.96) 99.

### Study approval

All animal experiments were conducted in compliance with the guidelines of the Institutional Animal Care and Use Committee of Nanyang Technological University (IACUC ARF-LKC/A18026, A19110) and SingHealth (2020/SHS/1593, 1594), Singapore, and the Guide for Care and Use of Laboratory Animals published by the US National Institutes of Health. Studies utilizing human tissues were approved by Nanfang Hospital, Southern Medical University (IRB: NFEC-2021-433). Written, informed consent was obtained from all study subjects before participation.

## Supplementary Material

Supplementary figures and tables.

## Abbreviations

AMY2: alpha-amylase
AP: acute pancreatitis
APAF: apoptotic peptidase activating factor
BMC: bone marrow cells
BMT: bone marrow transplantation
CARS: compensatory anti-inflammatory response syndrome
CCK: cholecystokinin
CCK1R: cholecystokinin Type 1 receptor
CCK2R: cholecystokinin Type 2 receptor
ChIP: chromatin Immunoprecipitation
Cyt C: cytochrome C
ERCP: endoscopic retrograde cholangiopancreatography
GCG: glucagon
H&E: hematoxylin and eosin
HOCOMOCO: HOmo sapiens COmprehensive MOdel Collection
HPaMEC: human pancreatic microvascular endothelial cell
IL: interleukin
INS: insulin
JNK: c-Jun N-terminal Kinase
KRT19: cytokeratin 19
LRG1: leucine-rich alpha-2 glycoprotein 1
MODS: multiorgan dysfunction syndrome
MPO: myeloperoxidase
PDL: pancreatic duct ligation
PKC: protein kinase C
SIRS: systemic inflammatory response syndrome
TGFβ: Transforming growth factor beta
TNF-α: tumour necrosis factor-alpha
Vegfa: vascular endothelial growth factor A
Vegfr: vascular endothelial growth factor receptor
WBC: white blood cell
