# Supplementary material for: LRG1 inhibition promotes acute pancreatitis recovery by inducing cholecystokinin Type 1 receptor expression via Akt
Source: Theranostics. 2025 Mar 18;15(10):4247–69. doi: 10.7150/thno.110116 (PMC11984386; doi:10.7150/thno.110116)
Supplement: Supplementary file 1 — Supplementary figures and tables. [file thnov15p4247s1.pdf]

Supplementary material

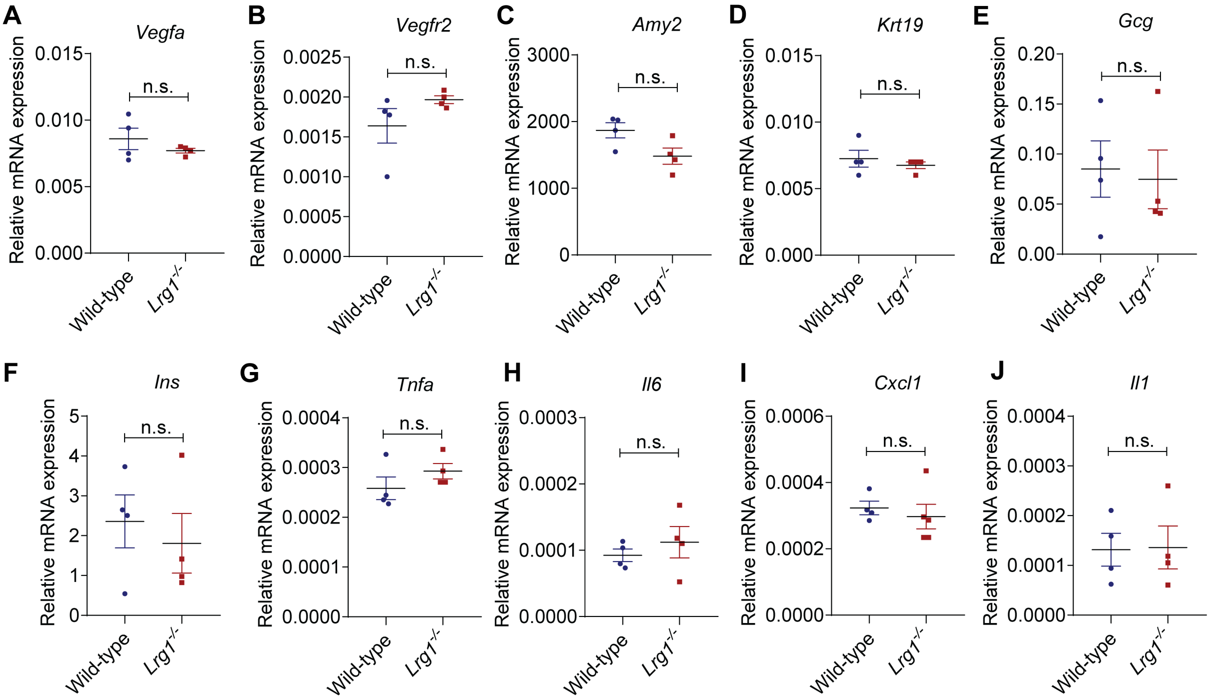

Supplementary Figure S1. LRG1 deficiency does not affect mRNA levels of pancreatic vascular, exocrine, endocrine, and inflammatory markers. qRT-PCR analysis of pancreatic **(A)** *Vegfa* **(B)** *Vegfr2* **(C)** *Amy2* **(D)** *Krt19* **(E)** *Gcg* **(F)** *Ins* **(G)** *Tnfa* **(H)** *Il6* **(I)** *Cxcl1* **(J)** *Il1* transcript levels in wild-type and *Lrg1*$^{-/-}$ mice. Data are represented as mean ± s.e.m. Significance was determined by unpaired, two-tailed Student's t-test of n ≥ 4 mice; n.s.: not significant, p > 0.05.

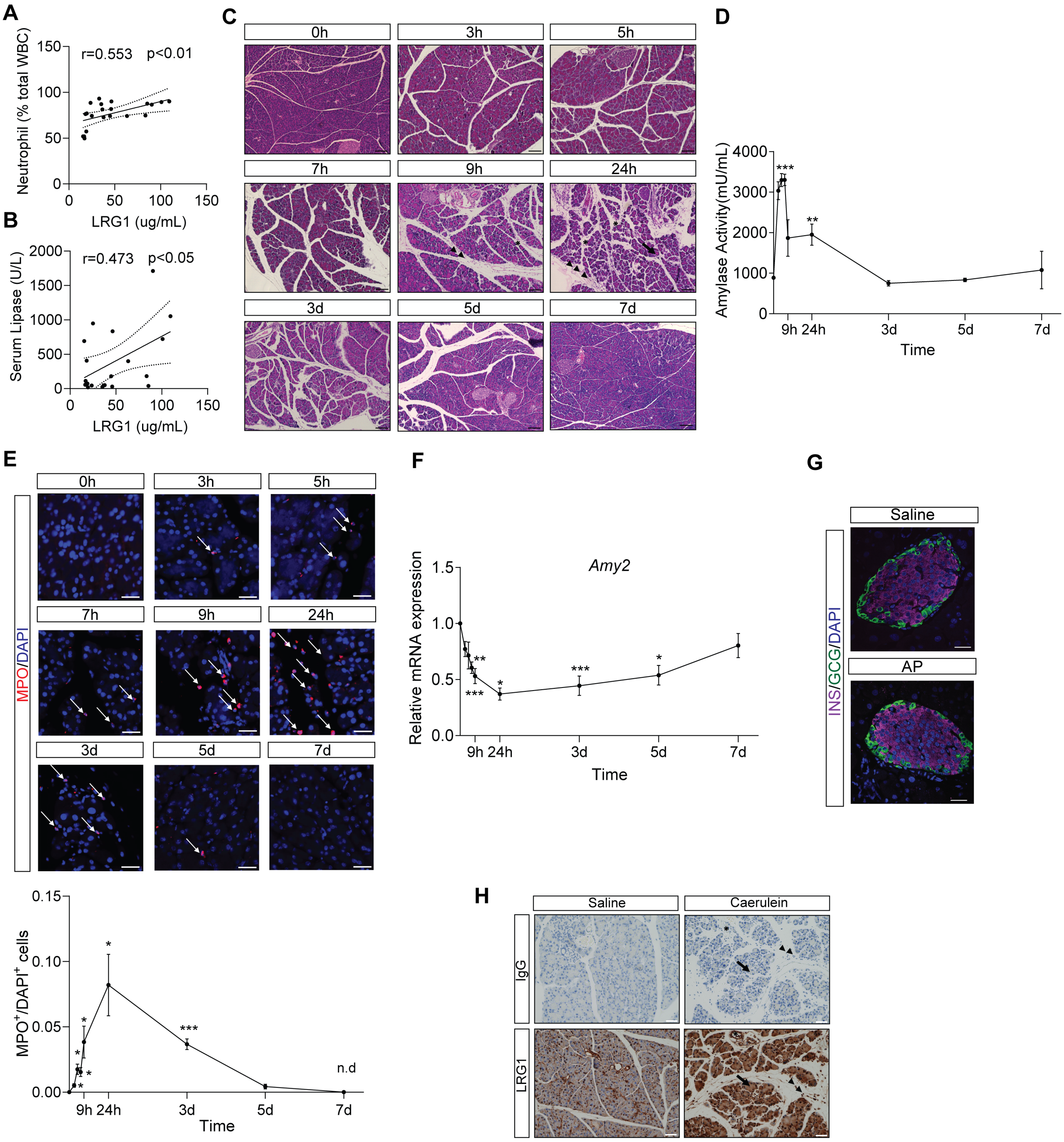

Supplementary Figure S2. Characterization of caerulein-induced AP mouse model. (A) Correlation analysis with regression line (95% confidence intervals) of serum LRG1 and neutrophil count as a percentage of total white blood cell (WBC) in AP patients. **(B)** Correlation analysis with regression line (95% confidence intervals) of serum LRG1 and serum lipase in AP patients. **(C)** H&E staining demonstrating changes in pancreatic tissue architecture in C57BL/6 mice at various time points during AP progression. Inter- and intralobular damage (asterisk), inflammatory cell infiltration (arrowhead), and edema (arrow) were highlighted in the pancreas. Scale bar: 100μm. **(D)** Serum amylase activity in C57BL/6 mice at various time points during AP progression. **(E)** Immunofluorescent staining against MPO (red) and DAPI (blue) (top) and quantification (bottom) of infiltrated MPO^{+} inflammatory cells (arrow). Scale bar: 25μm. **(F)** qRT-PCR analysis of pancreatic *Amy2* transcript levels at various time points during AP progression. **(G)** Immunofluorescence staining of insulin, INS (magenta) glucagon, GCG (green), and DAPI (blue) of C57BL/6 mouse pancreas from saline controls or 24 hours following the first caerulein injection, scale bar: 20μm. **(H)** Immunohistochemical detection against LRG1 (brown) or control IgG in the pancreas of saline or caerulein-treated C57BL/6 mice. Acinar cells (arrow), inflammatory cell infiltration (arrowhead), and vasculature (asterisk) were highlighted in the pancreas, scale bar: 50μm. All images are representative. Data are represented as mean ± s.e.m. Significance was determined by unpaired, two-tailed Student's t-test of n ≥ 4 mice; *: $p < 0.05$, **: $p < 0.01$, ***: $p < 0.001$, n.d.: no data available.

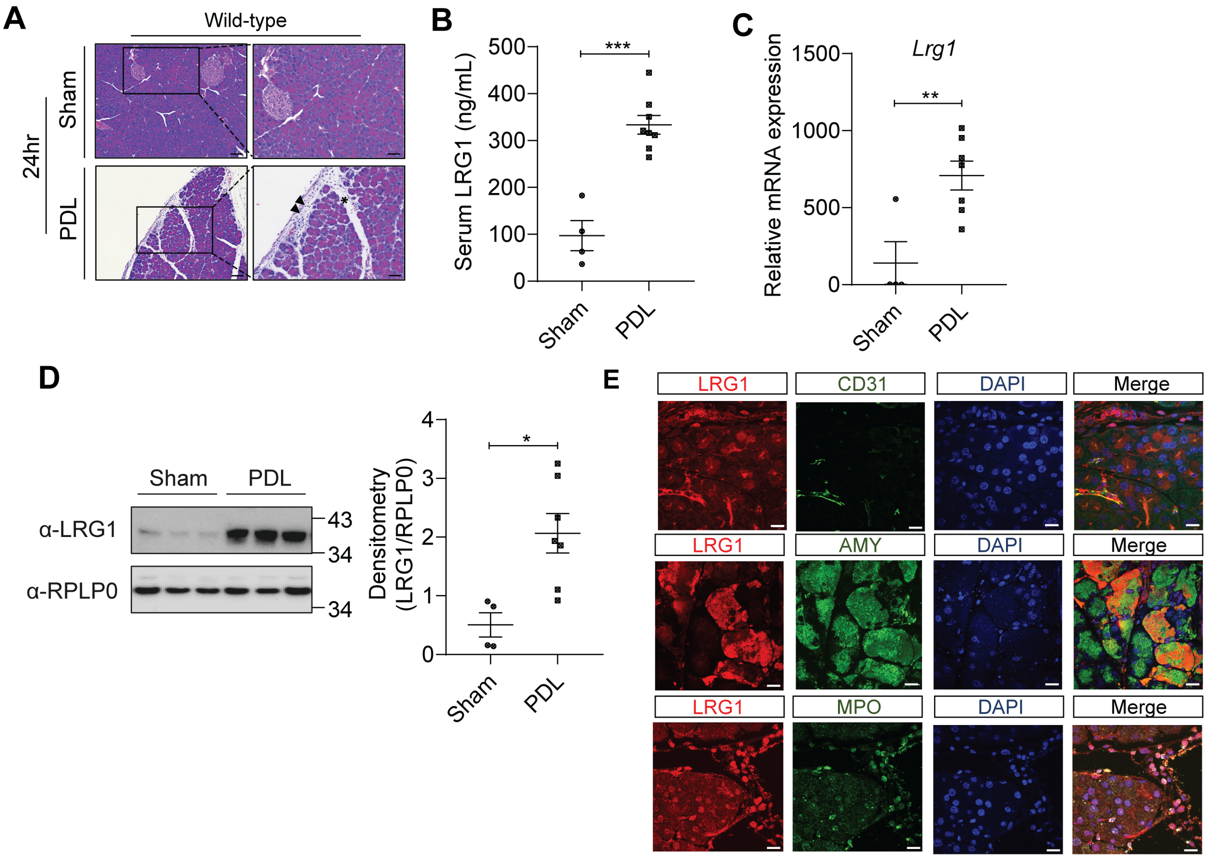

Supplementary Figure S3. Characterization of LRG1 in a pancreatic duct ligation AP mouse model. (A) H&E staining demonstrating changes in pancreatic tissue architecture in C57BL/6 mice subjected to pancreatic duct ligation (PDL) as compared to sham-operated controls. Inter- and intralobular space (asterisk) and inflammatory cell infiltration (arrowhead) were highlighted in the pancreas. Scale bar: 100μm, scale bar of boxed regions: 50μm. **(B)** ELISA analysis of serum LRG1 levels in mice subjected to PDL-induced AP. **(C)** qRT-PCR analysis of pancreatic *Lrg1* transcript levels and **(D)** Western blot (left) and densitometry analysis (right) showing pancreatic LRG1 protein levels in C57BL/6 mice subjected to PDL-induced AP or sham operation. **(E)** Immunofluorescent staining of LRG1 (red), CD31 or AMY or MPO (green), and DAPI (blue) in the pancreas of C57BL/6 mice subjected to PDL-induced AP. Scale bar: 10μm. All images are representative. Data are represented as mean ± s.e.m. Significance was determined by unpaired, two-tailed Student's t-test of $n \geq 4$ mice; *: $p < 0.05$, **: $p < 0.01$, ***: $p < 0.001$.

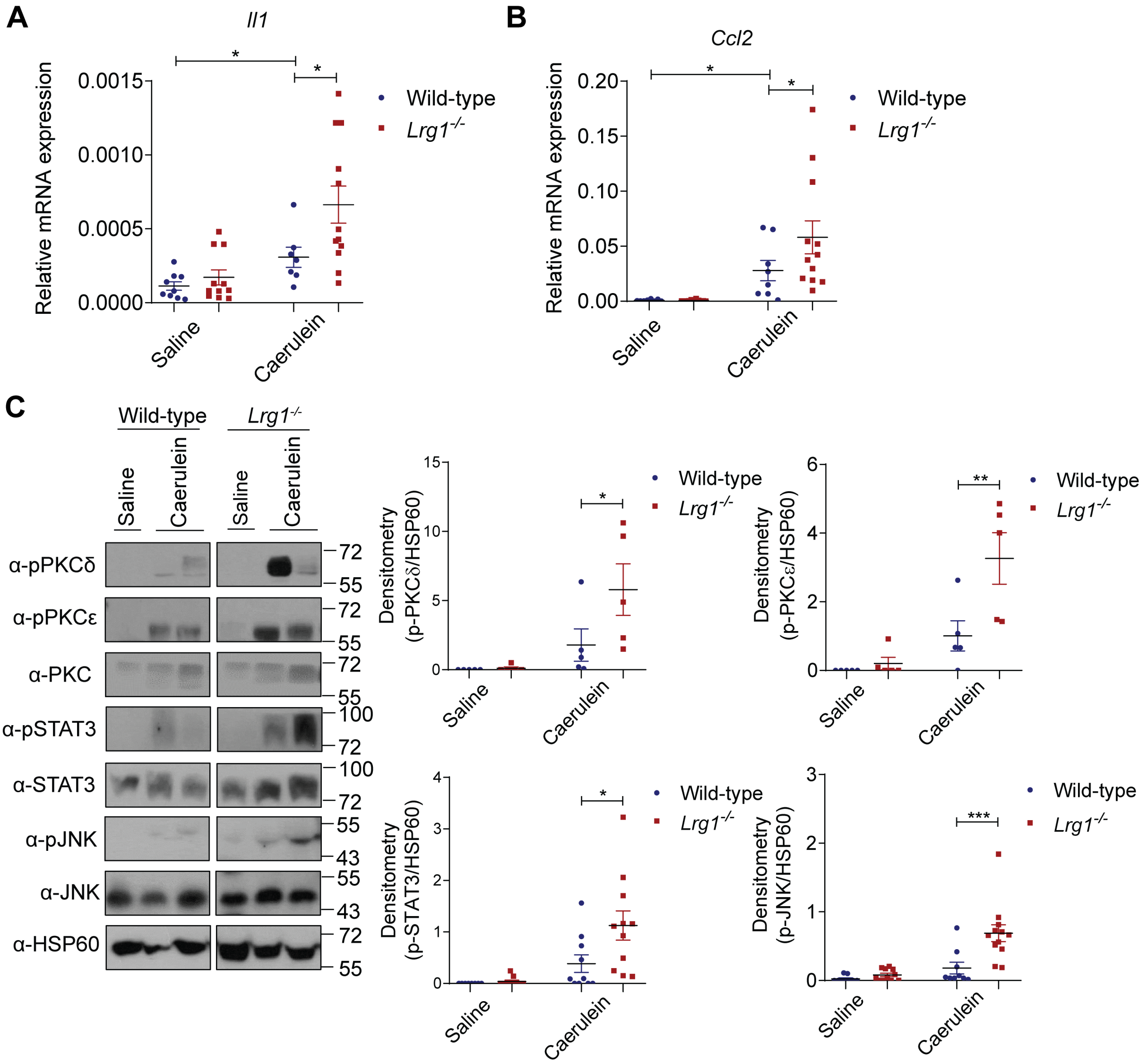

Supplementary Figure S4. Loss of LRG1 aggravates caerulein-induced AP. qRT-PCR analysis of the pancreatic **(A)** *Il1* and **(B)** *Ccl2* levels in wild-type and *Lrg1*-deficient mice 24 hours following AP induction. **(C)** Western blot (left) and densitometry analysis (right) illustrating phosphorylated and total levels of AP-associated signaling mediators PKC, STAT3, and JNK in wild-type and *Lrg1*$^{-/-}$ pancreas following AP induction. All images are representative. Data are presented as the mean ± s.e.m. Significance was determined by one-way ANOVA followed by Holm-Sidak's multiple comparison test or unpaired, two-tailed Student's t-test of n ≥ 6 mice; *: $p < 0.05$, **: $p < 0.01$, ***: $p < 0.001$.

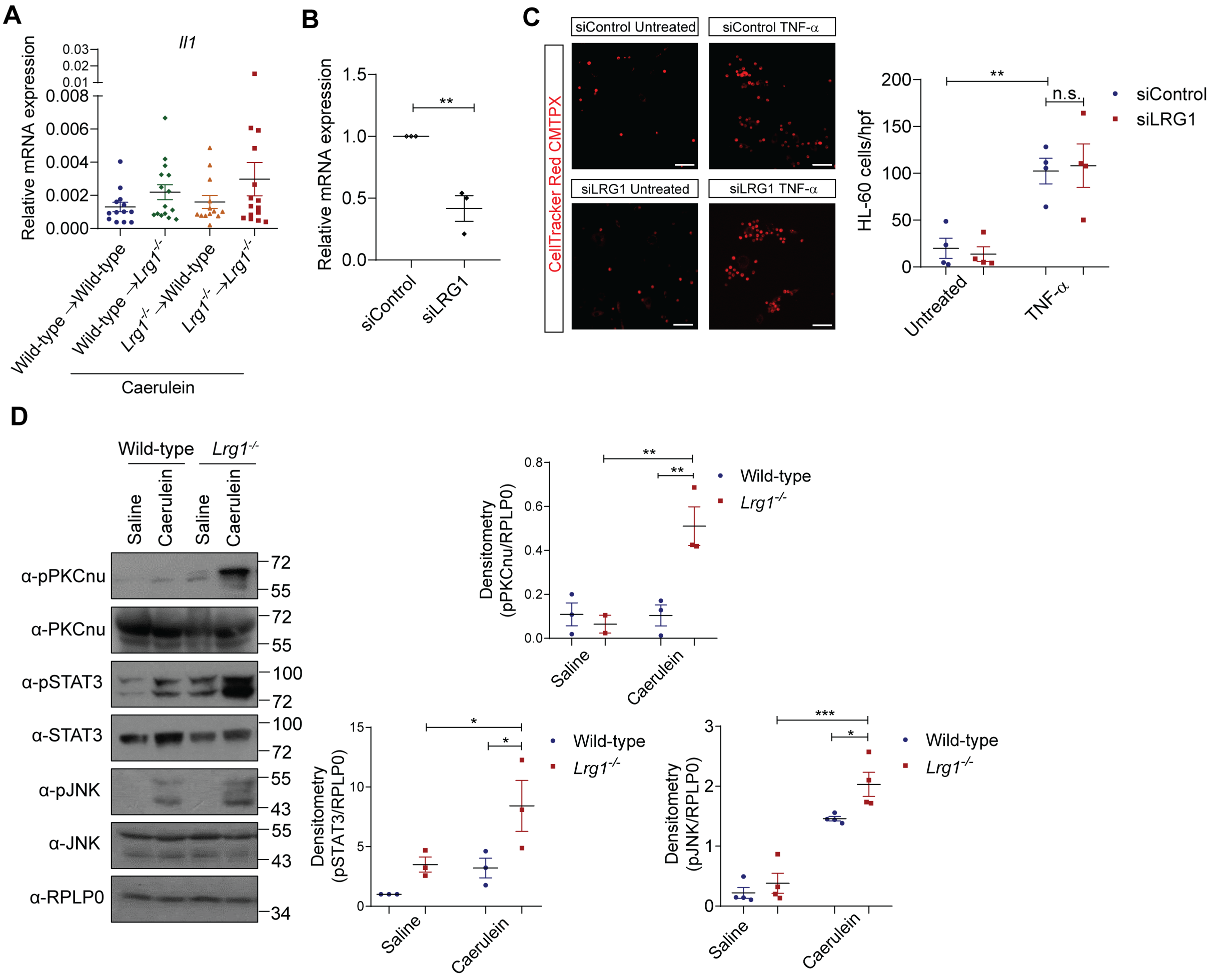

Supplementary Figure S5. **LRG1 deficiency in non-myeloid cells exacerbates AP development. (A)** qRT-PCR analysis of *Il1* mRNA levels in the pancreas of wild-type mice reconstituted with wild-type BMCs (Wild-type → Wild-type), *Lrg1*$^{-/-}$ mice reconstituted with wild-type BMCs (Wild-type → *Lrg1*$^{-/-}$), wild-type mice reconstituted with *Lrg1*$^{-/-}$ BMCs (*Lrg1*$^{-/-}$ →Wild-type) and *Lrg1*$^{-/-}$ mice reconstituted with *Lrg1*$^{-/-}$ BMCs (*Lrg1*$^{-/-}$ → *Lrg1*$^{-/-}$) at 24 hours post-AP induction. **(B)** qRT-PCR analysis of *LRG1* mRNA levels in HL-60 promyeloblast cells following siRNA-mediated silencing. **(C)** CellTracker Red CMTPX dye labelled images (left) and quantification (right) of TNFα-induced adhesion of *Lrg1*-knockdown HL-60 cells to human pancreatic microvascular endothelial cells. Scale bar: 75µm. **(D)** Western blot (left) and densitometry analysis (right) illustrating phosphorylated and total levels of AP-associated signaling mediators PKCnu, STAT3 and JNK in primary wild-type or *Lrg1*$^{-/-}$ acinar cells subjected to saline or caerulein treatment. All images are representative. Data are presented as the mean ± s.e.m. Significance was determined by one-way ANOVA followed by Holm-Sidak's multiple comparisons test of n ≥ 3 independent experiments or mice; *: $p < 0.05$, **: $p < 0.01$, ***: $p < 0.001$.

A

Gene Name	Ct value	∆Ct
**CCK1R**	29<Ct<31	9<∆Ct<12
**CCK2R**	17<Ct<19	1<∆Ct<2

B

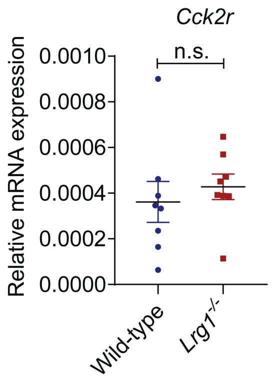

Supplementary Figure S6. CCKR expression in *Lrg1*-deficient pancreas. (A) Range of Ct and ∆Ct values of CCK1R and CCK2R in mouse pancreas tissue following qRT-PCR. **(B)** qRT-PCR analysis of *Cck2r* mRNA levels in the pancreas of wild-type and *Lrg1*$^{-/-}$ mice. Data are presented as the mean ± s.e.m. Significance was determined by unpaired, two-tailed Student's t-test of n ≥ 8 mice; n.s, not significant, $p > 0.05$.

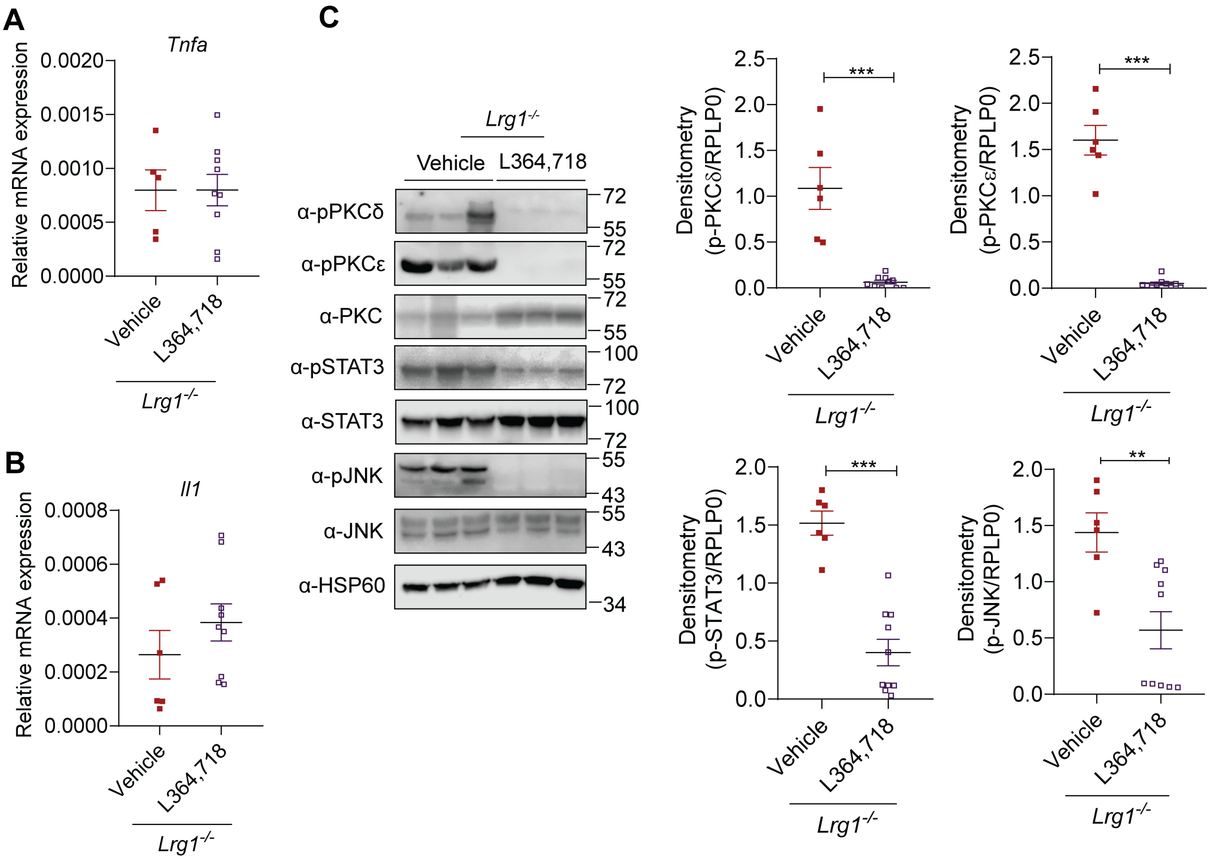

Supplementary Figure S7. CCK1R inhibition attenuates AP severity in *Lrg1*$^{-/-}$ mice. qRT-PCR analysis of inflammatory cytokine **(A)** *Tnfa* **(B)** *Il1* levels in vehicle or L364,718 treated *Lrg1*-deficient mice following the induction of AP. **(B)** Western blot (left) and densitometry analysis (right) illustrating phosphorylated and total levels of AP-associated signaling mediators PKC, STAT3, and JNK in the pancreas of vehicle or L364,718 treated *Lrg1*-deficient mice following the induction of AP. All images are representative. Data are presented as the mean ± s.e.m. Significance was determined by unpaired, two-tailed Student's t-test of n ≥ 5 mice; **: $p < 0.01$, ***: $p < 0.001$.

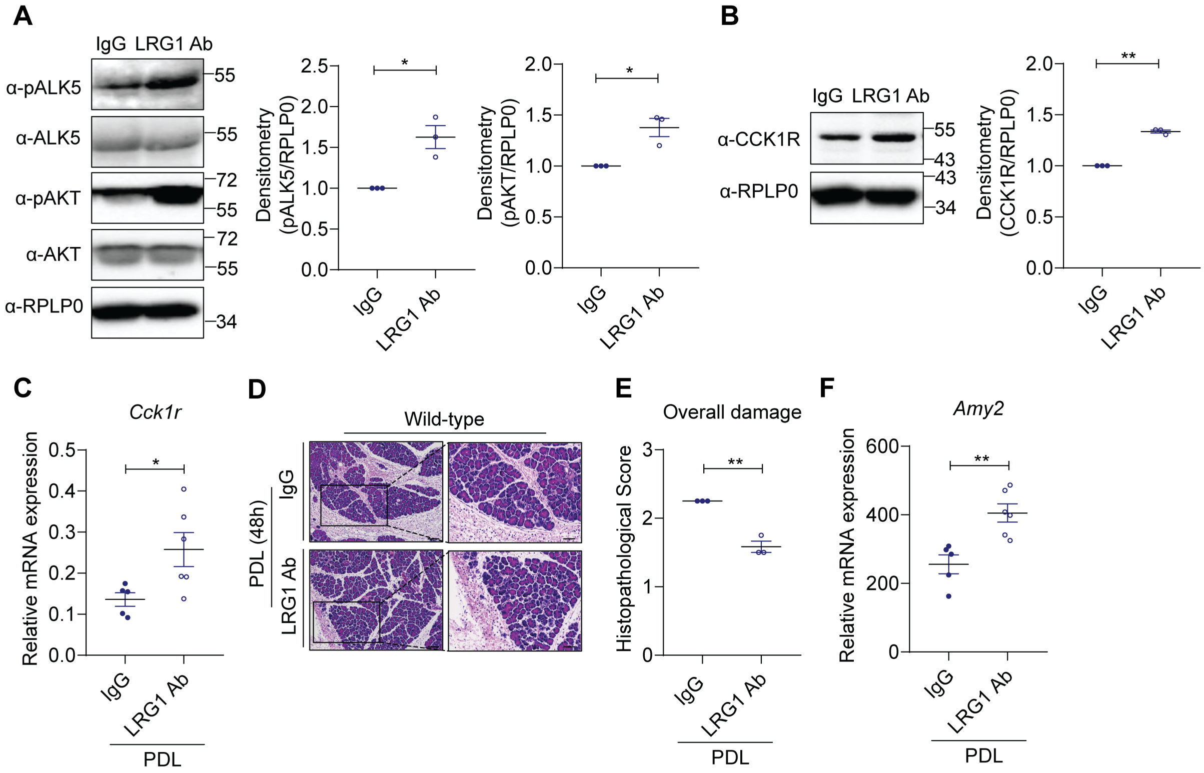

Supplementary Figure S8. LRG1 inhibition promotes pancreatic recovery in PDL-induced AP. (A) Western blot (left) and densitometry analysis (right) of phosphorylated and total levels of ALK5 and AKT protein in primary acinar cells isolated from wild-type mice subjected to IgG or LRG1 antibody treatment. **(B)** Western blot (left) and densitometry analysis (right) of CCK1R protein levels in primary acinar cells isolated from wild-type mice subjected to IgG or LRG1 antibody treatment. **(C)** qRT-PCR analysis of pancreatic *Cck1r* mRNA levels in AP mice treated with IgG or LRG1 antibody. **(D)** H&E staining and **(E)** histopathological grading of the pancreas of AP mice subjected to IgG or LRG1 antibody treatment. Scale bar: 100μm, Scale bar of boxed regions: 50μm. **(F)** qRT-PCR analysis of pancreatic *Amy2* levels in IgG or LRG1 antibody-treated mice. All images are representative. Data are presented as the mean ± s.e.m. Significance was determined by unpaired, two-tailed Student's t-test of $n \geq 3$ mice; *: $p < 0.05$, **: $p < 0.01$.

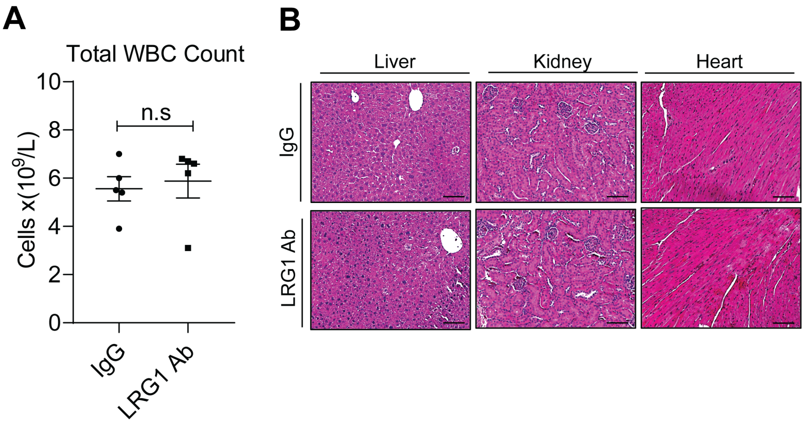

Supplementary Figure S9. No obvious toxicity following LRG1 antibody administration. (A) Total white blood cell (WBC) count in wild-type mice treated with IgG or LRG1 antibody. **(B)** H&E staining of key organs, liver, kidney and heart in wild-type mice following IgG and LRG1 antibody administration. Scale bar: 100µm. All images are representative. Data are presented as the mean ± s.e.m. Significance was determined by unpaired, two-tailed Student's t-test of n ≥ 3 mice; n.s. not significant.

Supplementary Table S1. Histopathological grading criteria

Grading Criteria	Score	Description
1) Inflammatory cell infiltration	G0	No abnormalities detected
	G1	Rare or around the periductal regions
	G2	Within the parenchyma (⩽25% of the lobules)
	G3	Within the parenchyma (>25-50% of the lobules)
	G4	Within the parenchyma (>50-75% of the lobules)
	G5	Within the parenchyma (>75% of the lobules)
2) Acinar lobular integrity damage	G0	No abnormalities detected
	G1	Minimal interlobular and intralobular space<br>No peri-parenchyma space
	G2	Mild interlobular and intralobular space<br>⩽ 25% peri-parenchyma space
	G3	Moderate interlobular and intralobular space<br>>25-50% peri-parenchyma space
	G4	Severe interlobular and intralobular space<br>>50-75% peri-parenchyma space
	G5	Severe interlobular and intralobular space<br>>75% peri-parenchyma space
3) Edema	G0	No abnormalities detected
	G1	Minimal edema, focally increased/expanded in interlobular septa
	G2	Mild edema, diffusely increased/expanded in interlobular septa
	G3	Moderate edema, diffusely increased/expanded in intralobular septa
	G4	Marked edema, diffusely increased/expanded in interacinar septa
	G5	Severe edema, diffusely increased/expanded in intercellular septa

Supplementary Table S2. qRT-PCR primer sequences

Gene	Forward Primer Sequence	Reverse Primer Sequence
**Mouse**		
Lrg1	5'-TGCACCTCTCGAGCAATCG-3'	5'-AGAGCATTGCGGGTCAGATC-3'
Vegfa	5'-GCGGATCAAACCTCACCAAA-3'	5'-TTCACATCTGCTGTGCTGTAGGA-3'
Vegfr2	5'-GGAGAAGAATGTGGTTAAGATCTG TGA-3'	5'-ACACATCGCTCTGAATTGTGTATACT-3'
Ins	5'-GGCTTCTTCTACACACCCA-3'	5'-CAGTAGTTCTCCAGCTGGTA-3'
Gcg	5'-ATCATTCCCAGCTTCCCAG-3'	5'-CGGTTCCTCTTGGTGTTCAT-3'
Amy2	5'-CAAAATGGTTCTCCCAAGGA-3′	5'-ACATCTTCTCGCCATTCCAC-3′
Krt19	5'-ACCCTCCCGAGATTACAACC-3′	5'-CAAGGCGTGTTCTGTCTCAA-3′
Cck1r	5'-TCTGGAACTCTACCAAGGAATC-3'	5'-GACCACAATGACAATGAGCATG-3'
Tnfa	5'-ATGAGCACAGAAAGCATGA-3′	5'-AGTAGACAGAAGAGCGTGG-3′
Nfkbia	5'-GTCTCCCTTCACCTGACCAA-3′	5'-CAGCAGCTCACGGAGGAC-3′
Il1	5'-CCAGCTTCAAATCTCACAGCAG-3′	5'-CTTCTTTGGGTATTGCTTGGGATC-3′
Il6	5'-GAAGTAGGGAAGGCCGTGG-3′	5'-CTCTGCAAGAGACTTCCATCCAGT-3′
Cxcl1	5'-TGAGCTGCGCTGTCAGTGCCT-3′	5'-AGAAGCCAGCGTTCACCAGA-3′
Ccl2	5'-GCTCAGCCAGATGCAGTTAA-3′	5'-TCTTGAGCTTGGTGACAAAAACT-3′
Il10	5'-ATAACTGCACCCACTTCCCA-3′	5'-GGGCATCACTTCTACCAGGT-3′
Ccnb	5'-CAGAGTTCTGAACTTCAGCCTG-3'	5'-TTGTGAGGCCACAGTTCACCAT-3'
Ccnd	5'-GCGTGCAGAAGGACATCCA-3'	5'-CACTTTTGTTCCTCACAGACCTCTAG-3'
Ccne	5'-TTGCACCAGTTTGCTTATGTTACA-3'	5'-AATGGTCAGAGGGCTTAGACG-3'
Rplp0	5'-AGATTCGGGATATGCTGTTGGC-3′	5'-TCGGGTCCTAGACCAGTGTTC-3′
